# Polymer Backbone Editing with Cyclopropenes via Olefin Metathesis

**DOI:** 10.1021/acsmacrolett.5c00734

**Published:** 2026-01-02

**Authors:** Jiyun Zhang, Will R. Gutekunst

**Affiliations:** † School of Chemistry and Biochemistry, 1372 Georgia Institute of Technology, 901 Atlantic Drive NW, Atlanta, Georgia 30318, United States

## Abstract

The limited ability to recycle or upcycle many commodity polymers with all carbon backbones poses a significant challenge to polymer chemists and society as a whole. In this work, sterically hindered cyclopropenes (**CPE**s) are used to promote ring-opening cross metathesis reactions with alkene-containing polymers to upgrade the original materials into functional copolymers through a formal backbone editing process. This polymer backbone editing process was able to achieve high conversion of the **CPE**s (>90%) and maintain reasonable postediting molecular weights (>22 kDa). The method was applied to different **CPE**s and olefin-containing polymers, resulting in changes in the chemical and thermal properties of the resulting copolymer materials. This work advances avenues for polymer upcycling processes, offering new directions for repurposing widely used olefinic polymers.

## Graphical abstract

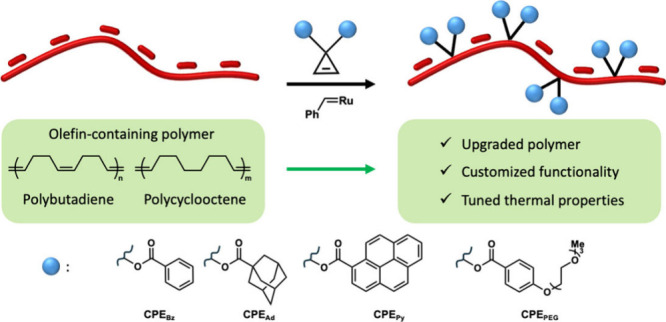

Polymers with olefinic backbones are widely used in everyday life and are a mainstay in a number of application areas. For example, the primary component used in tires, polybutadiene, is produced at a staggering rate of millions of metric tons annually.^1^ However, these materials pose a significant environmental challenge as they are not chemically recyclable, contributing to the issue of overflowing landfills and exacerbating the escalating environmental crisis.^2^ Consequently, there is a need to develop innovative chemical methods capable of facilitating upcycling processes, offering more sustainable pathways to address the end-of-life impact of these widely used olefinic polymers.^3–5^ Olefin metathesis is an attractive method to upgrade olefin-containing materials through the reorganization of alkene bonds in the polymer backbone in the presence of a metal carbene catalyst.^6–9^ Recent studies have explored the upcycling of polymers through ring-opening metathesis polymerization (ROMP) as a strategy for materials repurposing.^10^ Lloyd and Moore demonstrated the deconstruction and upcycling of poly­(dicyclopentadiene) via frontal copolymerization.^11^ Shieh and co-workers selectively installed cleavable bonds in the thermosets resulting in materials that can undergo controlled degradation to afford recyclable products with controlled size.^12^ Recently, a ring-opening cross metathesis strategy using norbornene monomers was reported by Foster and co-workers, leading to significant changes in the thermomechanical performance of the upcycled polymers.^13^ In this study, selective ring-opening cross metathesis of hindered cyclopropene derivatives is used to edit the backbones of olefin-containing polymers (Figure 1).

**Figure 1:**
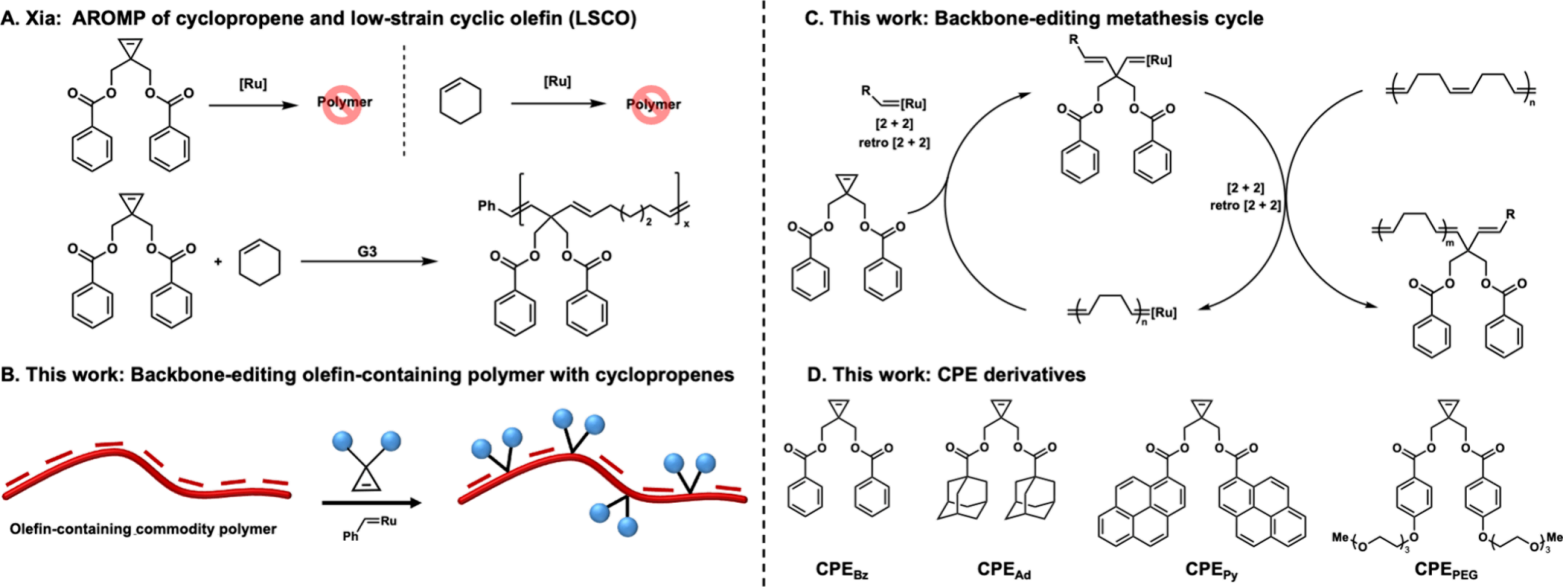
(A) Alternating ring-opening metathesis polymerization using sterically hindered cyclopropenes and low strain cyclic olefins. (B) Backbone editing of olefin-containing polymers with cyclopropenes. (C) Backbone editing metathesis cycle. (D) **CPE** derivatives used for backbone editing in this work.

Cyclopropenes (**CPE**s) are highly strained cyclic olefins (55 kcal/mol) which provide a strong thermodynamic driving force for ring-opening in olefin metathesis reaction in small molecule and macromolecular chemistry.^14–20^ **CPE**s were first shown by Schrock in 2006 to undergo ring-opening metathesis polymerization (ROMP) with molybdenum alkylidene initiators to give polymers with high monomer conversions and narrow dispersities.^17,21^ Later, Binder and Buchmeiser demonstrated unhindered 3,3-disubstituted cyclopropenes could also be polymerized using ruthenium-derived Grubbs-type initiators.^22,23^ In 2015, Xia and co-workers found that 3,3-disubstituted **CPE**s with increased steric bulk were unable to homopolymerize upon exposure to the Grubbs third generation initiator (**G3**), and only a single ring-opening reaction occurred (Figure 1A).^14,24,25^ Interestingly, this behavior was leveraged to achieve the highly alternating copolymerization when low-strain cyclic olefins (LSCOs) were combined with **CPE**s (Figure 1A).^14,24,26–31^ The LSCOs have little or no driving force for polymerization due to the lack of ring strain, but they can transiently ring-open due to the steric accessibility of the alkene. After ring-opening metathesis of the LSCO, another strained **CPE** can react to provide an overall alternating ring-opening metathesis polymerization (AROMP).

Building on Xia’s work use of unstrained cyclic olefins, we hypothesized that this method could be extended to the unstrained acyclic olefins in a polymer backbone to affect a formal editing of the polymer chain (Figure 1B).^32,33^ The envisioned backbone editing metathesis cycle begins with the ring-opening of **CPE** by the Grubbs catalyst (Figure 1C). Since the resulting ruthenium carbene is incapable of further homopropagation, the ensuing metathesis reaction would occur at an unhindered olefin on the polymer backbone. While this initially cleaves the polymer backbone, further reaction with **CPE** and additional polymer chains would lead to an overall stitching process. The metathesis reactions then continue until all the cyclopropenes are consumed and fully inserted into the polymer chain. This approach to backbone modification is expected to lead to randomly incorporated CPE units, in contrast to the study by Foster where added norbornene monomers have the potential to homopolymerize and generate blocky segments in the polymer chain.^13^

Initial studies toward backbone editing with **CPE**s examined polycyclooctadiene (**PCOD**) as a model system, which is identical to linear 1,4-polybutadiene. High molecular weight **PCOD** (*M*_n_ = 118 kDa, *Đ* = 1.83, Figure S1) containing 88% *trans* and 12% *cis* alkene isomers was prepared through ROMP of cyclooctadiene with Grubbs second generation initiator (G2) following standard procedures (Table S1). A **CPE** derivative with two benzoate substituents (**CPE**_**Bz**_) was prepared via a previously reported four-step synthesis from trimethylsilyl acetylene and was used for initial investigations.^34^ To examine the viability of this backbone editing process, **CPE**_**Bz**_ and **PCOD** (1:5 mol ratio) were reacted with 1.2 mol % of the Grubbs third generation catalyst (**G3**) in THF (0.1 M) at room temperature for 20 h, then quenched with ethyl vinyl ether (EVE). Analysis of the crude ^1^H NMR (Figure 2) revealed high conversions (>95%) of **CPE**_**Bz**_ by monitoring the alkene singlet at 7.36 ppm and noticeable broadening of the aromatic proton resonances (7.4–8.1 ppm). After precipitation of the sample in methanol, the aromatic benzoate proton resonances were retained, supporting the successful stitching of **CPE**_**Bz**_ into the **PCOD** backbone. DOSY NMR was performed on **PCOD**-*co*-**CPE**_**Bz**_ and confirmed the presence of a single diffusing polymeric species, consistent with the successful stitching of **CPE** units into the polymer chain (Figure S3). Integration of isolated proton resonances from **PCOD** (allylic CH_2_, 2.03 ppm) and **CPE**_**Bz**_ (phenyl CH, 8.02 ppm) were used to calculate the ratio of **CPE**_**Bz**_ and COD units (1:5) on the purified polymer backbone, which aligned with the initial feed ratio 1:5 (Figure S2). With a starting **PCOD** molecular weight of 118 kDa and 20 h of reaction time, the postedited polymer molecular weight (*M*_n_) was measured by size exclusion chromatography (SEC) to be 45 kDa (Table S2, entry 1). The structure of the backbone editing product was further confirmed through independent copolymerization between COD and **CPE**_**Bz**_ (Table S3, entry 1, Figure S4). The copolymerization yielded an identical ^1^H NMR spectrum compared with the backbone edited **PCOD**.

**Figure 2:**
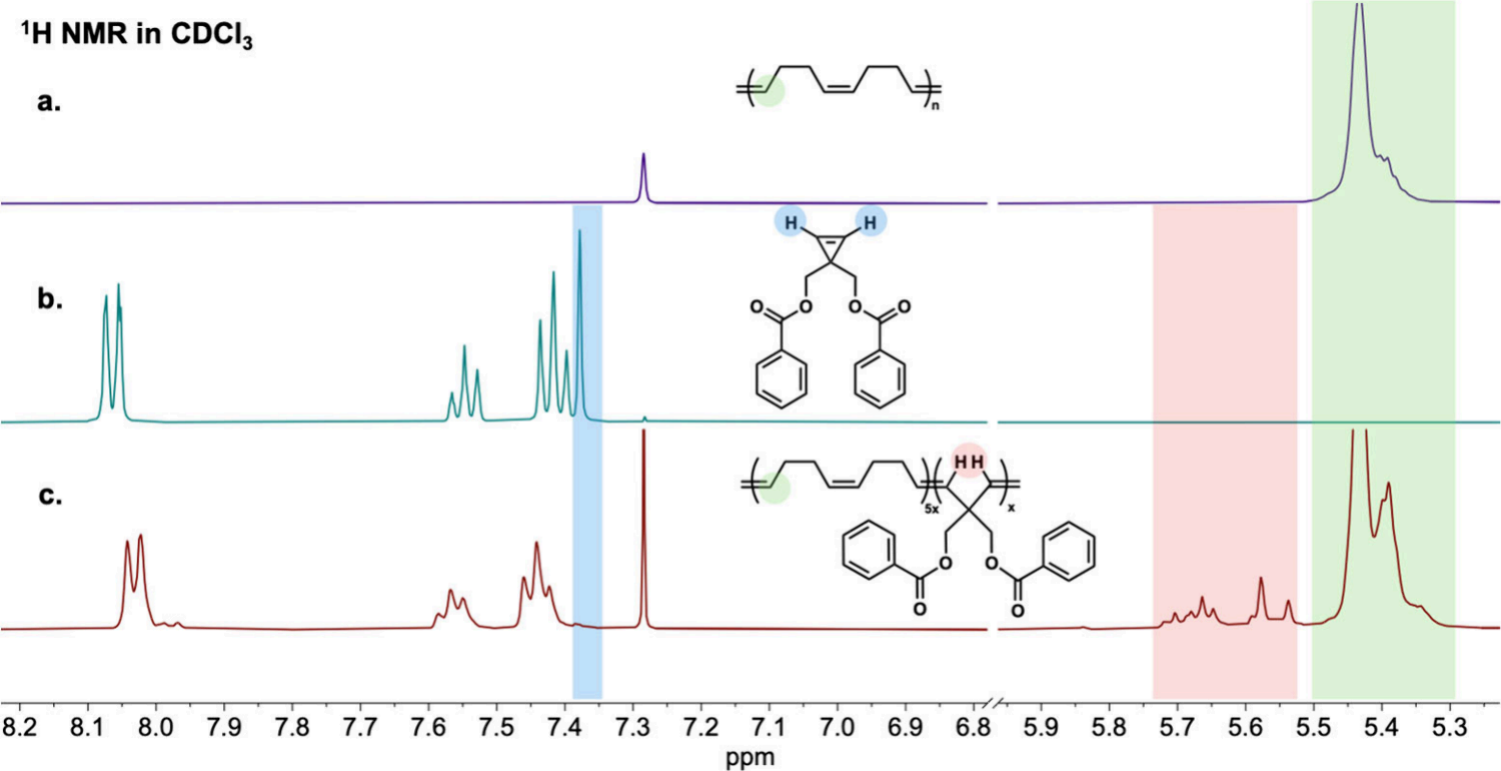
^1^H NMR for backbone editing of **PCOD**-*co*-**CPE**Bz: (a) ^1^H NMR of **PCOD**; (b) ^1^H NMR of **CPE**Bz monomer; (c) Crude ^1^H NMR of the backbone editing reaction mixture.

While some decrease in molecular weight was anticipated due to initial chain scission events after **CPE** ring-opening, the observed reduction was more than estimated from the catalyst loading or deviations related to the use of polystyrene standards in the SEC calibration. A known backbiting process for **PCOD** is the formation of a low-strain molecule, *trans*, *trans*, and *trans*-1,5,9-cyclododecatriene (CDT). Isomerization of the **PCOD** backbone through chain transfer results in a primarily *trans*-**PCOD** backbone, which leads to the formation of CDT.^35^ The formation of CDT, tracked by ^1^H NMR integration of the peak at 5.0 ppm, is a side product that contributes to the reduced yield and molecular weight of the edited polymer along with potential formation of other low molecular weight cyclic oligomers. Upon the introduction of **G3**, an equilibrium is established between the formation of low-strained cyclic oligomers/CDT through backbiting and the productive backbone editing process. The equilibrium can be shifted toward productive polymer editing by increasing the concentration of the backbone editing reaction (Table S4). In addition, higher loadings of **G3** increase the scission of the polymer backbone which lowers the postedited molecular weights of **PCOD**. As a result, low catalyst loading is crucial for maintaining the desired molecular weights of **PCOD**-*co* **-CPE**_**Bz**_ (Table S2, entries 5–9). To optimize the backbone editing process, different reaction concentrations and **G3** loadings were evaluated. Increasing the reaction concentration to 0.6 M in THF suppressed the formation of CDT, which in turn increases the final polymer molecular weight to 34 kDa (Table S4).^35^ **G3** loadings were screened at 0.24, 0.6, 0.9, 1.2 mol % and 2.4 mol % at 0.6 M. Incomplete conversion of **CPE**_**Bz**_ was observed below 0.9 mol % (Table S2, entries 5 and 6), and decreases in postedit molecular weight were observed above 0.9 mol % catalyst (Table S2, entries 8 and 9). The concentrations of 0.6 M and 0.9 mol % **G3** were found to be optimal for backbone editing for minimizing CDT formation while maintaining high conversions (>95%) and *M*_n_ (39 kDa) of the edited **PCOD** (Table S2, entry 7).

The substituents on **CPE**s can be readily modified to afford diverse backbone-edited polymers. To investigate the impact that the **CPE** has on the material properties of the edited polymer, a series of **CPE**s with adamantane (**CPE**_**Ad**_), oligoethylene glycol (**CPE**_**PEG**_), and pyrene (**CPE**_**Py**_) substituents were synthesized and evaluated using the optimized conditions for backbone editing with **CPE**_**Bz**_. Using a 1:5 molar ratio of **CPE** to backbone alkene, **PCOD**-*co*-**CPE**_**Ad**_ was obtained with a molecular weight of 38 kDa (Table 1, entry 2) and >95% conversion of **CPE**_**Ad**_, behaving similarly to **PCOD**-*co*-**CPE**_**Bz**_. Under the same reaction conditions, **PCOD**-*co*-**CPE**_**Py**_ was obtained with a *M*_n_ of 37 kDa (Table 1, entry 3) and 52% conversion of **CPE**_**Py**_, possibly due to increased steric hindrance of the pyrene substituent, and **PCOD**-*co*-**CPE**_**PEG**_ was obtained with a *M*_n_ of 37 kDa (Table 1, entry 4) and 83% **CPE** conversion. By decreasing the **CPE** to **PCOD** alkene ratio in the reaction mixture, the degree of editing of the polymer backbone editing could readily be tuned, with the final copolymer composition in good agreement with feed ratios (Table 1, entries 5–8). In all cases, the **CPE** derivatives were successfully inserted into the polymer backbone with high conversions and maintained similar molecular weights to the reactions at higher **CPE** loadings (Table 1).

**Table 1:**
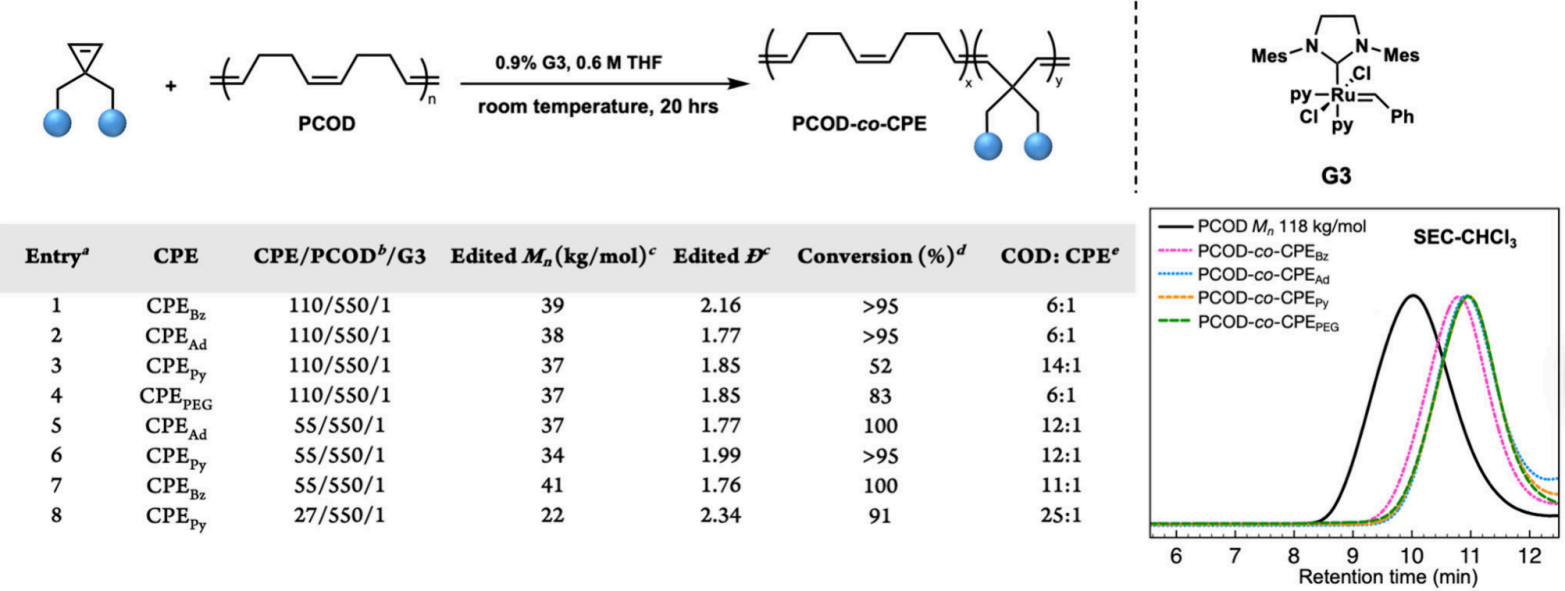


a Backbone editing conditions: 0.9 mol % **G3**, **CPE** and **PCOD** in THF at room temperature for 20 h.

b **PCOD** has a *M*_n_ of 118 kDa and a dispersity of 1.83. [**PCOD**] indicates the alkene repeat units within **PCOD**.

c Number average molecular weights and dispersities after backbone editing determined by CHCl_3_ SEC calibrated using polystyrene standards of the precipitated reaction mixture.

d Conversions were determined by ^1^H NMR of the crude reaction mixture in CDCl_3_.

e The ratio of COD alkenes and **CPE** repeat units was determined by ^1^H NMR spectroscopy of the precipitated reaction mixture; Bottom left corner: Overlaid SEC traces of **PCOD** and backbone-edited **PCOD** with different **CPE**s.

To explore an additional material for backbone editing, polycyclooctene (**PCOE**, *M*_n_ = 200 kDa, *M*_w_ *=* 348 kDa, *Đ* = 1.74) was prepared through ROMP of cyclooctene with **G3** in 0.5 M CH_2_Cl_2_ following standard procedures (Table S5). Initial experiments for **PCOE** backbone editing were performed with **CPE**_**Bz**_ and **PCOE** (1:5 mol ratio) with **G3** in THF under N_2_ at room temperature for 20 h under optimized conditions for **PCOD**. Interestingly, these reaction conditions were ineffective for **PCOE** backbone editing due to solubility challenges in THF and led to low conversions of **CPE**_**Bz**_. Switching the solvent to chloroform and increasing the concentration of the reaction mixture were found to increase the **CPE** conversions to 79% at 0.8 M (Table S6, entries 1–7). Catalyst loading was also examined, and a decreased catalyst percentage was found to help maintain the edited *M*_n_ as expected (Table S6, entries 7–10). 2.4 mol % **G3** was identified as the optimal catalyst loading for maximizing *M*_n_ while maintaining high (93%) **CPE** conversion. Given the absence of CDT formation in the PCOD modification, the isolated yield of the final copolymers also increased (**PCOE**-*co*-**CPE**_**Bz**_ yield of 87% compared to a **PCOD**-*co* **-CPE**_**Bz**_ yield of 53%, Table S7). Using these optimized conditions, **CPE** derivatives **CPE**_**Ad**_, **CPE**_**Py**_, and **CPE**_**PEG**_ were used to edit the backbone of **PCOE** (Table 2). All **CPE**s were able to achieve high conversions in the upcycling process and maintain *M*_n_s above 30 kDa, though slightly reduced conversions were again observed for **CPE**_**Py**_ (Table 2, entry 3). The edited *M*_n_ is 73 kDa for **PCOE**-*co*-**CPE**_**PEG**_ and is higher than the usual **PCOE** backbone editing (Table 2, entry 4). Fractionation occurred during purification of **PCOE**-*co*-**CPE**_**PEG**_ due to the high solubility of PEG groups, leading to an increased *M*_n_ value of the isolated edited polymer.

**Table 2:**
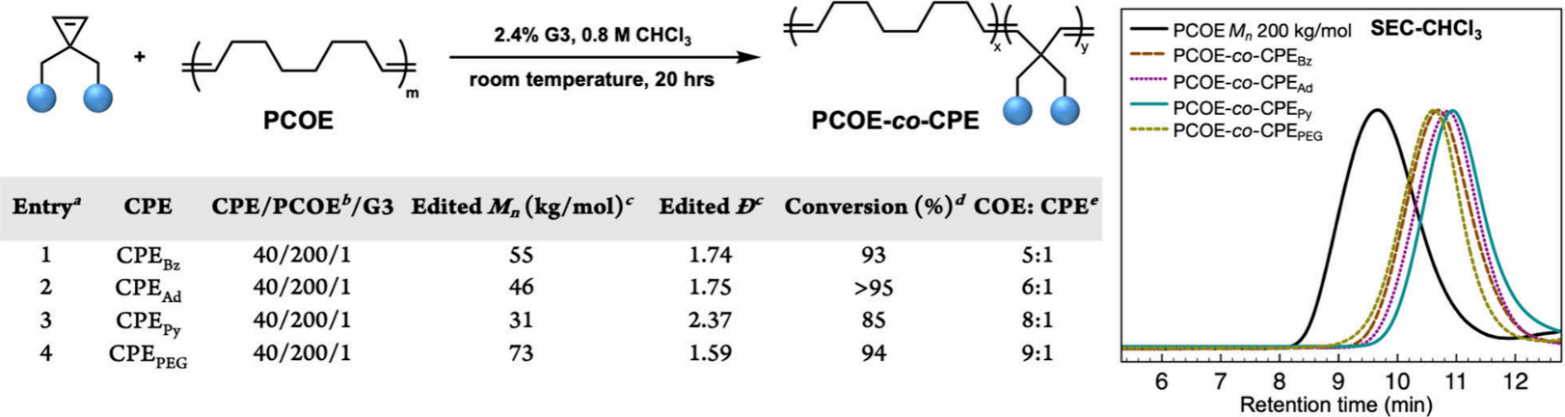


a Backbone editing conditions: 2.4 mol % **G3**, **CPE** and **PCOE** in CHCl_3_ at room temperature for 20 h.

b **PCOE** has a *M*_n_ value of 200 kDa and a dispersity of 1.74. [**PCOE**] indicates the alkene repeat units within **PCOE**.

c Number average molecular weights and dispersities after backbone editing determined by CHCl_3_ SEC calibrated using polystyrene standards of precipitated reaction mixture.

d Conversions were determined by ^1^H NMR of crude reaction mixture in CDCl_3_.

e The ratio of COE units and **CPE** units is determined by ^1^H NMR of the precipitated reaction mixture. On the right: SEC of **PCOE** and backbone-edited **PCOE** with different **CPE**s.

Thermal analysis of the backbone-edited **PCOD**-**CPE** copolymers by differential scanning calorimetry (DSC) was used to understand changes in the thermal properties of the edited materials (Table S8). Compared to the unedited **PCOD** which has a melting temperature (*T*_m_) of 68 °C and a crystallization temperature *T*_c_ = 50 °C, the **PCOD**-*co*-**CPE**_**Ad**_ (**PCOD**:**CPE**_**Ad**_ ratio of 12:1) results in a reduction of both the *T*_m_ (20 °C) and *T*_c_ (−6 °C), as well as broadening of both of these transitions. Similarly, **PCOD**-*co*-**CPE**_**Py**_ (**PCOD**:**CPE**_**Py**_ ratio of 14:1) reduced the *T*_m_ and *T*_c_ to 24 and −27 °C, respectively (Figure 3). By reducing the amount of CPE_py_ in **PCOD** **CPE**_**Py**_ backbone editing, the *T*_m_ = 27 °C and *T*_c_ = 5 °C both increase compared to the **PCOD** **CPE**_**Py**_ backbone editing with higher **CPE**_**py**_ ratio, indicating that by changing the **PCOD**:**CPE** ratio, the material properties of the resulting polymer can be successfully tuned (Table S8). In addition, with a 6:1 **PCOD**:**CPE**_**PEG**_ ratio, the *T*_m_ and *T*_c_ were lowered to 18 and −11 °C, respectively. For **PCOD**-*co*-**CPE**_**Bz**_ (**PCOD**:**CPE**_**Bz**_ 11:1), the *T*_m_ = 21 °C and *T*_c_ = 3 °C was also lowered compared to unedited **PCOD**. The decrease in crystallinity of the copolymer materials with increased editing is expected, as the **CPE** segments act as defects in the **PCOD** chain and reduce segment lengths available for crystallization. Analogous changes in the thermal properties of the **PCOE**-edited materials followed the same trends (Table S8).

**Figure 3:**
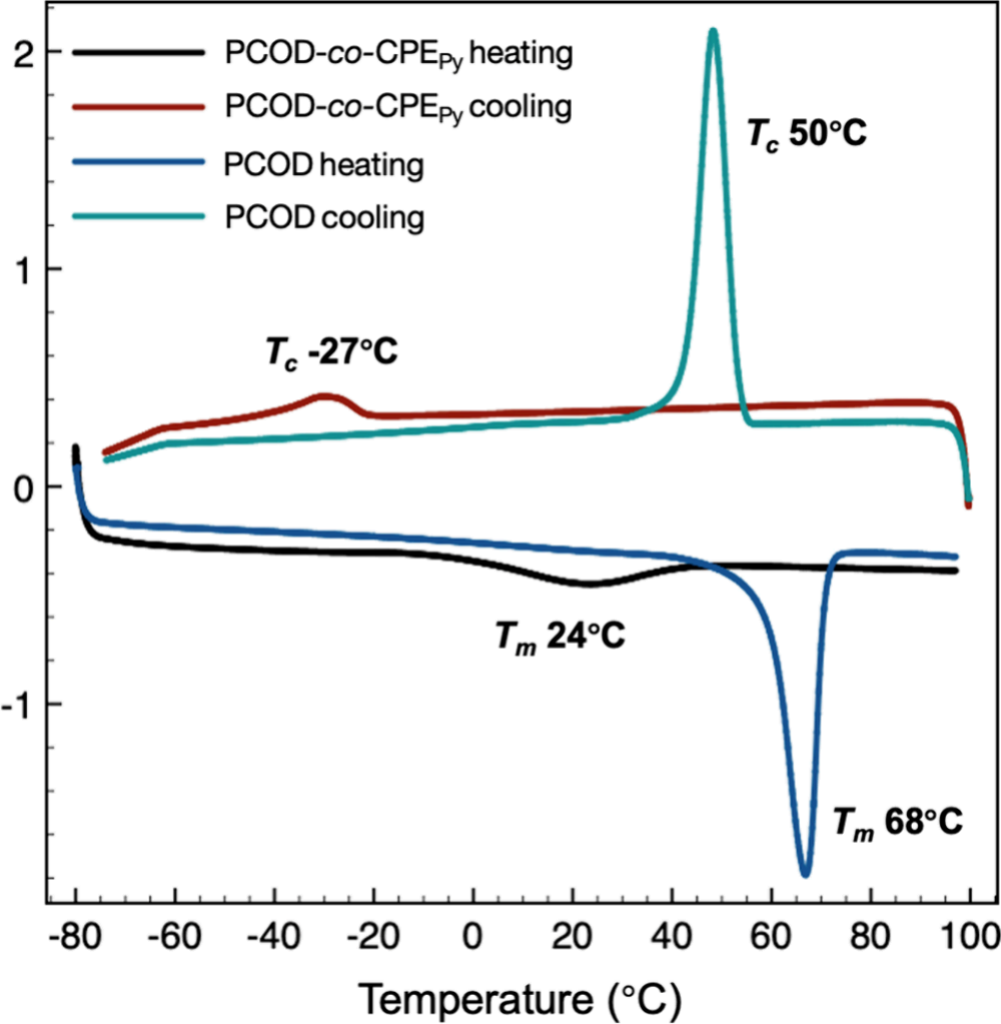
DSC traces for **PCOD** and **PCOD**-*co*-**CPEPyrene** (Table 1, entry 3).

In addition to influencing the thermal properties of the final material, the introduction of **CPE** groups modified the chemical properties of the edited materials. The introduction of PEG chains increased the hydrophilicity of the copolymers as reduced water contact angles were observed on **PCOE** and **PCOE**-*co*-**CPE**_**PEG**_ films (Figure S7). Whereas the **PCOE** film has an average contact angle of 88.0°, **PCOE**-*co*-**CPE**_**PEG**_ (**PCOE** to **CPE**_**PEG**_ ratio of 9:1) has an average contact angle of 77.1°. Additionally, the inclusion of **CPE_Py_** introduces new fluorescence behavior to both **PCOD** and **PCOE** (Figure S8), underscoring the impact of the CPE group incorporation on the overall material properties.

In conclusion, a new method of inserting target functionalities into olefinic polymer backbones with **CPE**s via ring-opening cross metathesis was developed. The sterically hindered **CPE**s were stitched into the backbone of **PCOD** and **PCOE** with Grubbs third generation catalyst to achieve upgraded polymers with various functionalities while maintaining high conversions and moderate molecular weights. The thermal and chemical properties of **PCOD** and **PCOE** were shown to be tunable through the insertions of the **CPE**s, suggesting new opportunities for upcycling existing rubber materials.

## Supplementary Material



## Notes

Additional experimental details and methods, including ^1^H NMR spectra for all polymers and CPE derivatives and DSC and GPC curves (PDF)
