# Supplementary material for: Polymer Backbone Editing with Cyclopropenes via Olefin Metathesis
Source: ACS Macro Lett. 2026 Jan 2;15(1):196–201. doi: 10.1021/acsmacrolett.5c00734 (PMC12825371; doi:10.1021/acsmacrolett.5c00734)
Supplement: Supplementary file 1 [file mz5c00734_si_001.pdf]

Supporting Information

# Polymer Backbone Editing with Cyclopropenes via Olefin Metathesis

Jiyun Zhang[1] and Will R. Gutekunst[1,*]

1. School of Chemistry and Biochemistry, Georgia Institute of Technology, 901 Atlantic Drive NW, Atlanta, Georgia 30318, United States

**Table of Contents**

General Procedures----------1

$^1$H NMR ($CDCl_3$) of **PCOD** ----------2

COD Polymerization Conditions----------2

$^1$H NMR of **PCOD** precipitated calculation with **CPE$_{Bz}$** ----------3

DOSY plot ----------4

**PCOD** Backbone-editing Condition Screening Table----------4

COD and COE copolymerization with **CPE$_{Bz}$** ----------5-6

**PCOD** Backbone-editing with CDT: **CPE** Ratio----------7

COE Polymerization Conditions----------8

**PCOE** Backbone-editing Condition Screening Table----------8

Mass yield of polymers ----------9

DSC characterization of backbone-edited **PCOD** and **PCOE** with **CPEs**----------9

Contact angle measurements for hydrophilicity----------10

Fluorescent images of **PCOD-*co*-CPE$_{Py}$** ----------10

Experimental Procedures for Functional Groups Synthesis ----------11

$^1$H and $^{13}$C NMR Spectra for **CPE** Derivatives----------12-14

$^1$H NMR ($CDCl_3$) of **PCOE** ----------16

Backbone-editing Mechanism and Theoretical Mn calculations----------17-18

Kinetics Data on **CPE$_{Bz}$** **PCOD** and **PCOE** Backbone Editing ----------19

Impact on **PCOD** $M_n$ with by varying catalyst loading and the Mn of pre-edited **PCOD** ----------19

$^1$HNMR Confirmation of **CPE$_{Bz}$** Incorporation on **PCOE** ----------20

$^1$H NMR of **PCOE** conversion and precipitated calculation with **CPE$_{Bz}$** ----------21

GPC of **PCOE** backbone editing crude and precipitation comparison----------22

GPC of **PCOD** backbone editing crude and precipitation comparison----------23

**General Procedures.** All reactions were carried out under a nitrogen atmosphere with dry solvents using anhydrous conditions unless otherwise stated. Dry, degassed DCM and THF were obtained from a JC Meyer solvent purification system. $CDCl_3$ was stored under 4 Å molecular sieves. Yields refer to chromatographically and spectroscopically($^1$H-NMR) homogeneous materials. Reactions were monitored by thin layer chromatography (TLC) carried out on 0.25 mm E. Merck silica gel plates (60F-254) using UV light as the visualizing agent and basic aqueous potassium permanganate (KMnO4), and heat as developing agents. SiliCyclicsilica gel (60, particle size 0.043–0.063 mm) was used for flash column chromatography. NMR spectra were recorded on Bruker Avance 400, 500 or 700 MHz instruments and calibrated using residual undeuterated solvent as an internal reference ($CHCl_3$ at 7.26 ppm $^1$H NMR, 77.16 ppm $^{13}$C NMR). The following abbreviations (or combinations thereof) were used to explain the multiplicities: s = singlet, d = doublet, t = triplet, q = quartet, m = multiplet, br = broad. Polymer samples were analyzed using a Tosoh EcoSEC HLC 8320GPC system with TSKgel SuperHZ-L columns eluting $CHCl_3$ containing 0.25% $NEt_3$ at a flow rate of 0.45 mL/min. All number-average molecular weights and dispersities were calculated from refractive index chromatograms using PStQuick Mp-M polystyrene standards. Differential scanning calorimetry (DSC) analyses were measured on a DSC 3+ STARe system (Mettler Toledo). The reported data were obtained from the third heating cycle at a heating rate of 10 °C/min.

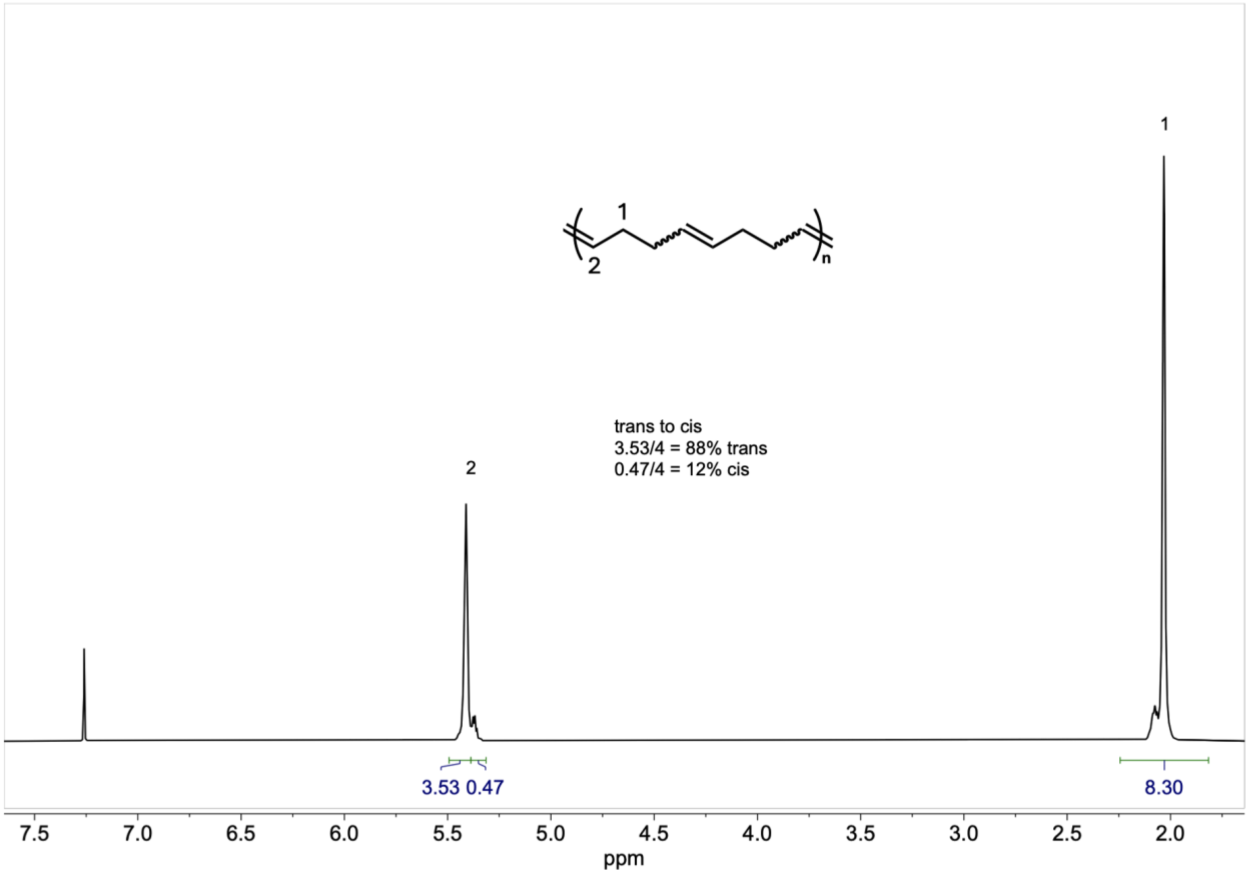


**Figure S1. $^1$H NMR ($CDCl_3$) of PCOD.**

**Table S1. Cyclooctadiene Polymerization via ROMP Condition Screening**

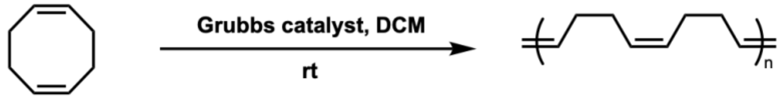


| Entry[a] | Catalyst | M/I[e] | [M] (mol/L) | Time (h) | Conversion (%)[b] | $M_{n,\ theo}$[c] (kg/mol) | $M_{n,\ SEC}$[d] (kg/mol) | $Đ$[d] |
|---|---|---|---|---|---|---|---|---|
| 1 | Grubbs 3 | 300 | 3 | 21 | ≥98 | 33 | 63 | 1.88 |
| 2 | Grubbs 3 | 900 | 3 | 19 | ≥98 | 99 | 91 | 1.91 |
| 3 | Grubbs 3 | 1200 | 3 | 19 | ≥98 | 132 | 110 | 1.90 |
| 4 | Grubbs 2 | 300 | 4 | 22 | ≥98 | 33 | 60 | 2.10 |
| 5 | Grubbs 2 | 900 | 4 | 22 | ≥98 | 99 | 97 | 2.11 |
| 6 | Grubbs 2 | 1200 | 4 | 22 | ≥98 | 132 | 110 | 2.04 |

[a]Polymerization of cyclooctadiene was carried out with 0.924 mmol scale under $N_2$. [b]Conversions were determined by $^1$H NMR of crude reaction mixture. [c]$M_{n,theo}$ = n × conv. × M(cyclooctadiene). [d]Number average molecular weights and dispersities were determined by size-exclusion chromatography using polystyrene standards. [e]Degree of polymerization = $[Monomer]_0/[Initiator]_0$.

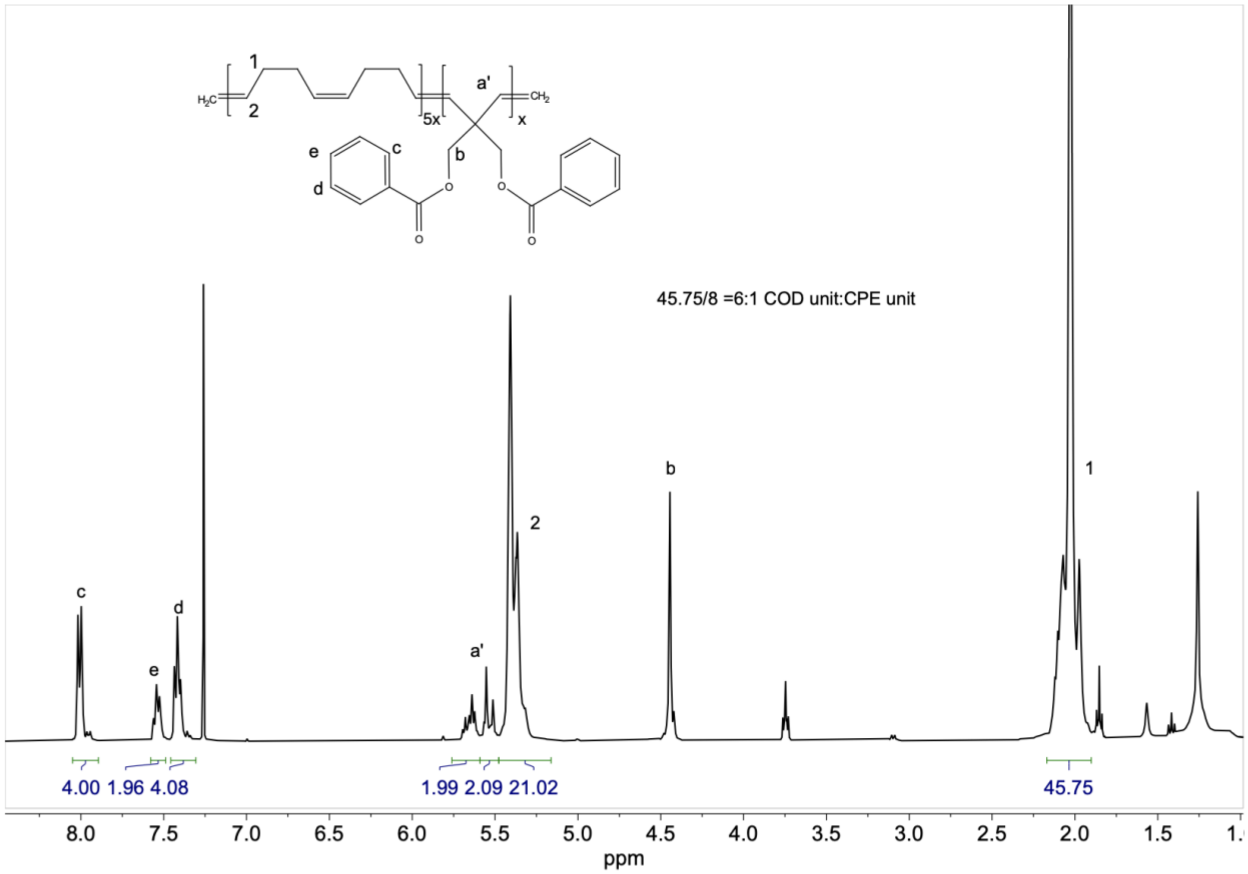


**Figure S2. Pure $^{1}$H NMR spectra for CPE-Bz edited PCOD conversion and COD:CPE-Bz calculations.**

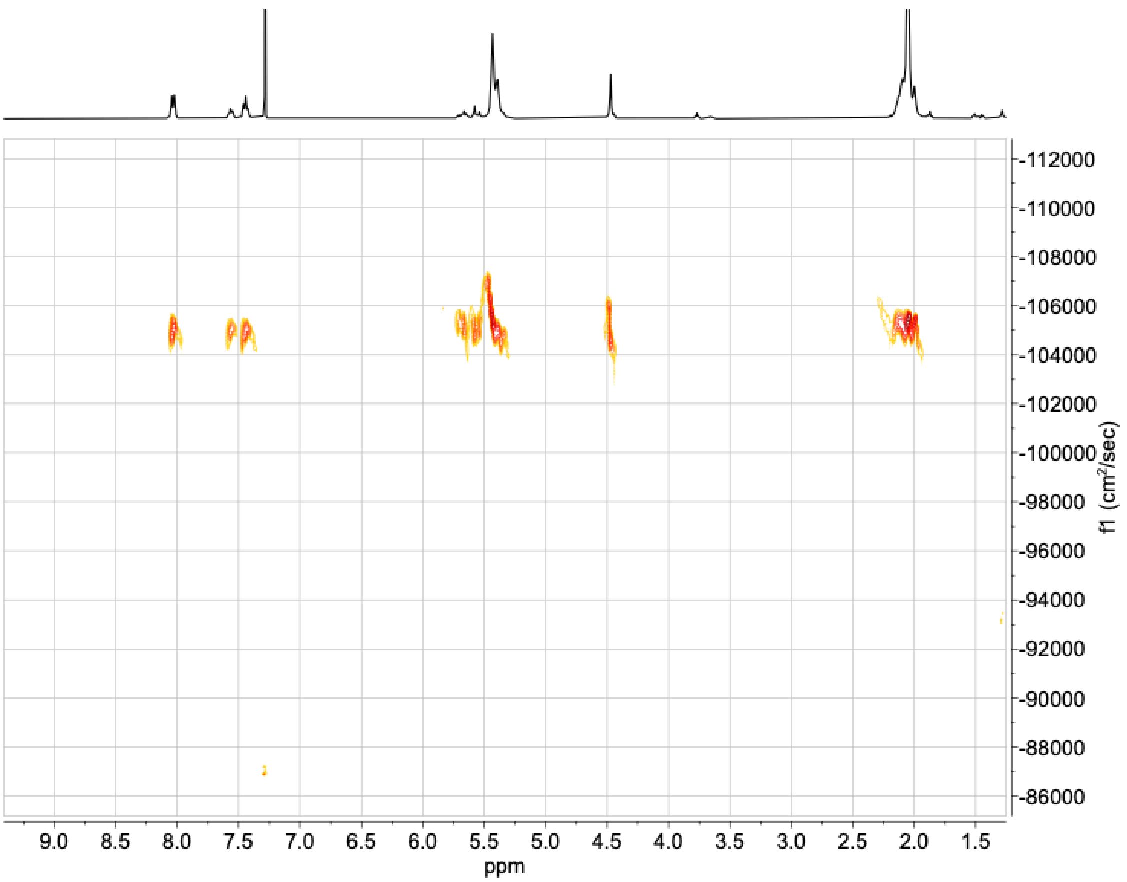


**Figure S3. DOSY plot for Table 1 entry 1.**

**Table S2. Backbone editing of PCOD with CPE-Bz screening results.**

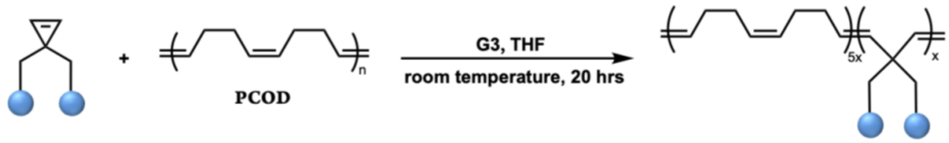


| Entry[a] | CPE | CPE/PCOD[b]/G3 | Catalyst percent (%) | [M] (mol/L) | Edited $M_n$ (kg/mol)[c] | Edited $Đ$[c] | Conversion (%)[d] | COD: CPE[e] |
|---|---|---|---|---|---|---|---|---|
| 1 | CPE-Bz | 110/550/1.32 | 1.2 | 0.1 | 45 | 1.68 | >95 | 5:1 |
| 2 | CPE-Bz | 110/550/1.32 | 1.2 | 0.2 | 27 | 1.54 | >95 | 6:1 |
| 3 | CPE-Bz | 110/550/1.32 | 1.2 | 0.4 | 25 | 1.68 | 92 | 6:1 |
| 4 | CPE-Bz | 110/550/1.32 | 1.2 | 0.6 | 34 | 1.78 | 91 | 6:1 |
| 5 | CPE-Bz | 110/550/0.26 | 0.24 | 0.6 | 52 | 1.64 | 37 | 9:1 |
| 6 | CPE-Bz | 110/550/0.66 | 0.6 | 0.6 | 37 | 1.65 | 73 | 4:1 |
| 7 | CPE-Bz | 110/550/1 | 0.9 | 0.6 | 39 | 2.16 | >95 | 6:1 |
| 8 | CPE-Bz | 110/550/1.32 | 1.2 | 0.6 | 30 | 2.20 | >95 | 3:1 |
| 9 | CPE-Bz | 110/550/2.64 | 2.4 | 0.6 | 23 | 1.98 | >95 | 3:1 |

[a]Backbone editing conditions: G3 catalyst, CPE and PCOD in THF at room temperature for 20 hours. [b]PCOD has a $M_n$ of 118 kg/mol and a dispersity of 1.83. [PCOD] indicates the COD units within PCOD, each COD unit has 2 olefins. [c]Number average molecular weights and dispersities after backbone editing determined by $CHCl_3$ size-exclusion chromatography (SEC) calibrated using polystyrene standards of precipitated reaction mixture. [d]Conversions were determined by $^1H$ NMR of crude reaction mixture in $CDCl_3$. [e]The ratio of COD units and CPE units are determine by $^1H$ NMR of precipitated reaction mixture.

**Table S3. COD and COE copolymerization with CPE-Bz.**

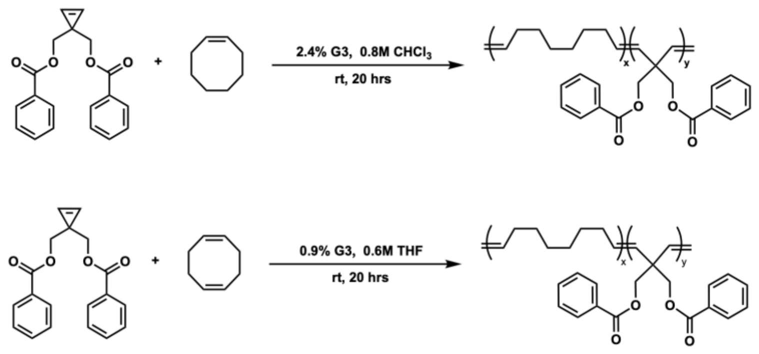


| Entry | CPE | Cyclic monomer | CPE/Monomer/G3 | Catalyst percent (mol %) | [M] (mol/L) | Edited $M_n$ (kg/mol) | Edited $Đ$ | Conversion (%) | Monomer: CPE |
|---|---|---|---|---|---|---|---|---|---|
| 1 | $CPE_{Bz}$ | COD | 100/500/1.2 | 0.9 | 0.6 | 56 | 1.84 | 94 | 6:1 |
| 2 | $CPE_{Bz}$ | COE | 100/500/2.4 | 2.4 | 0.8 | 45 | 1.91 | 92 | 5:1 |

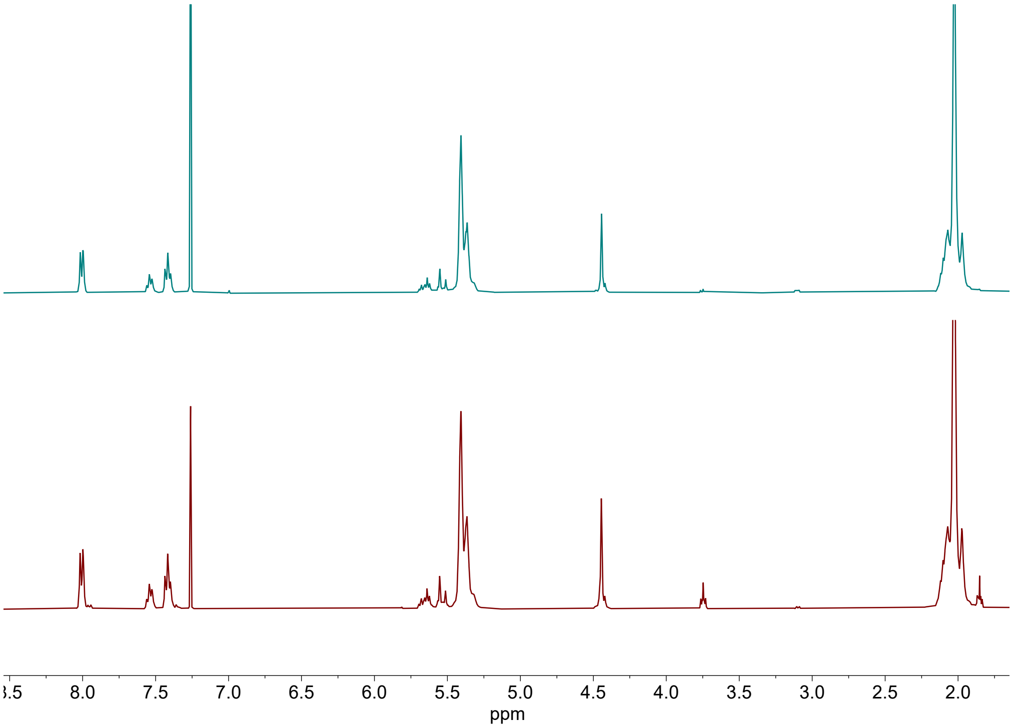


**Figure S4. $^{1}$H NMR COD $CPE_{Bz}$ copolymerization (top, Table S3, entry 1) and PCOD-*co*-$CPE_{Bz}$ (bottom, Table 1, entry 1) after precipitation.**

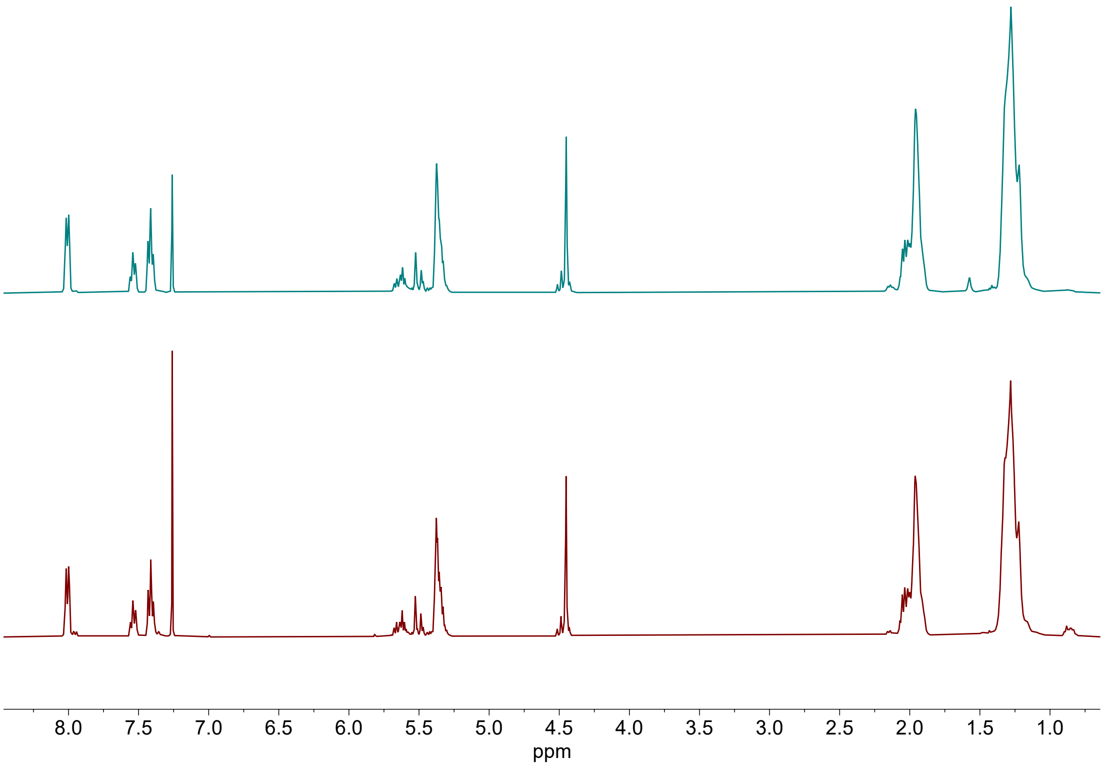


**Figure S5.** $^{1}$**H NMR COE CPE$_{Bz}$ copolymerization (top, S25, entry 2) and PCOE-*co*-CPE$_{Bz}$ (bottom, table 2, entry 1) after precipitation.**

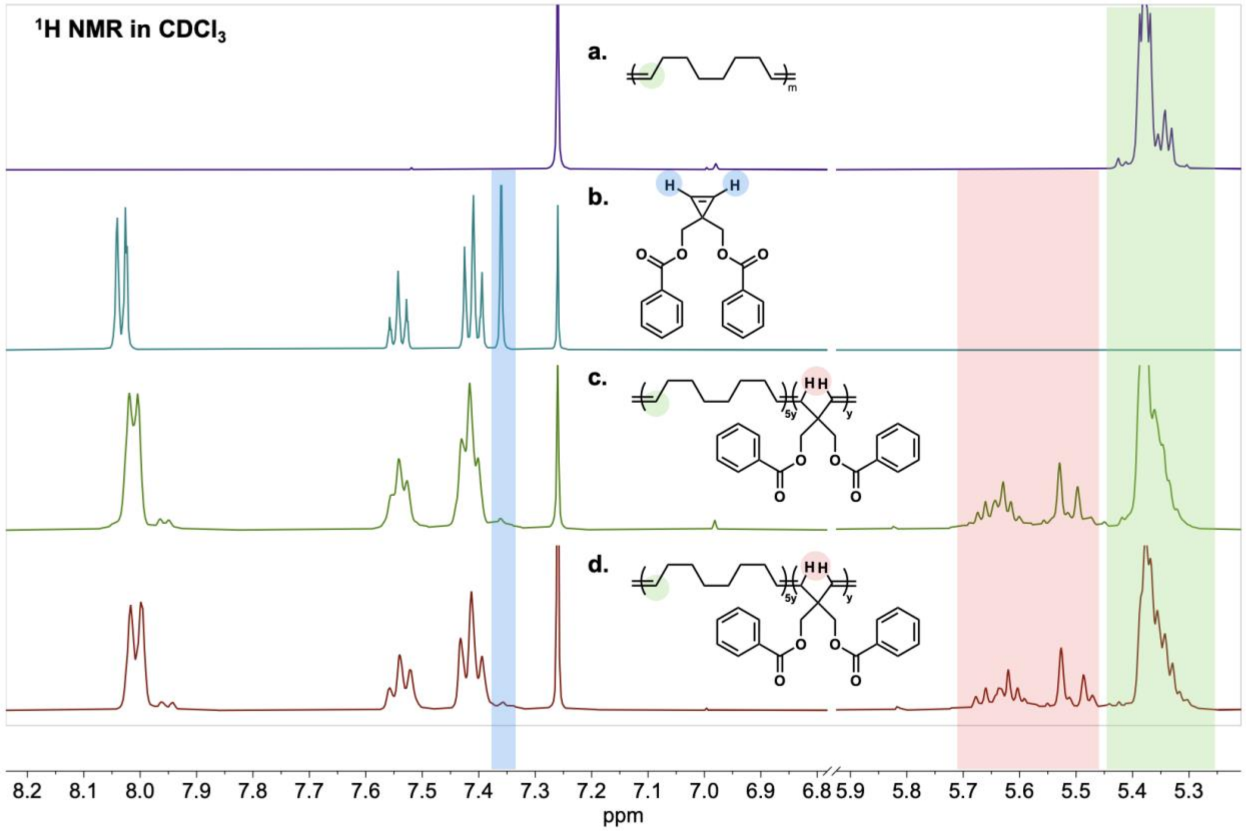


$^a$PCOE, $^b$CPE-benzoyl monomer, $^c$crude backbone editing reaction mixture, $^d$precipitated backbone editing reaction mixture.

**Figure S6. Crude $^1$H NMR spectra for CPE-Bz edited PCOE conversion.**

**Table S4. Backbone editing of PCOD with CPE-Bz concentration screening with CDT:CPE ratio.**

| Entry[a] | CPE | CPE/PCOD[b]/G3 | Catalyst percent (%) | [M] (mol/L) | Edited $M_n$ (kg/mol)[c] | Edited $Đ$[c] | Conversion (%)[d] | COD: CPE[e] | CDT: CPE[f] |
|---|---|---|---|---|---|---|---|---|---|
| 1 | CPE-Bz | 110/550/1.32 | 1.2 | 0.1 | 45 | 1.68 | >95 | 5:1 | 1.4:1 |
| 2 | CPE-Bz | 110/550/1.32 | 1.2 | 0.2 | 27 | 1.54 | >95 | 6:1 | 1.1:1 |
| 3 | CPE-Bz | 110/550/1.32 | 1.2 | 0.4 | 25 | 1.68 | 92 | 6:1 | 0.64:1 |
| 4 | CPE-Bz | 110/550/1.32 | 1.2 | 0.6 | 34 | 1.78 | 91 | 6:1 | 0.42:1 |

$^a$Backbone editing conditions: G3 catalyst, CPE and PCOD in THF at room temperature for 20 hours. $^b$PCOD has a $M_n$ of 118 kg/mol and a dispersity of 1.83. [PCOD] indicates the COD units within PCOD, each COD unit has 2 olefins. $^c$Number average molecular weights and dispersities after backbone editing determined by $CHCl_3$ size-exclusion chromatography (SEC) calibrated using polystyrene standards of precipitated reaction mixture. $^d$Conversions were determined by $^1$H NMR of crude reaction mixture in $CDCl_3$. $^e$The ratio of COD units and CPE units are determined by $^1$H NMR of precipitated reaction mixture. $^f$The ratio of CDT units and CPE units are determined by $^1$H NMR of precipitated reaction mixture.

## Table S5. Cyclooctene Polymerization via ROMP Condition Screening

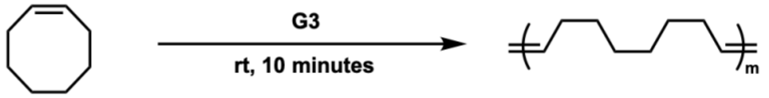


| Entry[a] | M/I/BHT | [M] (mol/L) | Time (min) | Solvent | Conversion (%)[b] | $M_{n,\,theo}$[c] (kg/mol) | $M_{n,\,SEC}$[d] (kg/mol) | Đ[d] |
|---|---|---|---|---|---|---|---|---|
| 1 | 600/1/0 | 0.5 | 10 | DCM | ≥98 | 65 | 125 | 1.76 |
| 2 | 1200/1/0 | 0.5 | 10 | DCM | ≥98 | 130 | 51 | 3.70 |
| 3 | 1200/1/12 | 0.5 | 10 | DCM | ≥98 | 130 | 207 | 1.73 |
| 4 | 600/1/0 | 0.5 | 10 | THF | ≥98 | 65 | 130 | 1.77 |
| 5 | 1200/1/0 | 0.5 | 10 | THF | ≥98 | 130 | 153 | 1.78 |
| 6 | 1200/1/12 | 0.5 | 10 | THF | ≥98 | 130 | 173 | 1.77 |

[a]Polymerization of cyclooctadiene was carried out with 0.924 mmol scale under $N_2$. [b]Conversions were determined by $^1H$ NMR of crude reaction mixture. [c]$M_{n,theo}$ = n × conv. × M(cyclooctene). [d]Number average molecular weights and dispersities were determined by size-exclusion chromatography using polystyrene standards.

## Table S6. Backbone editing of PCOE with CPE-Bz screening results.

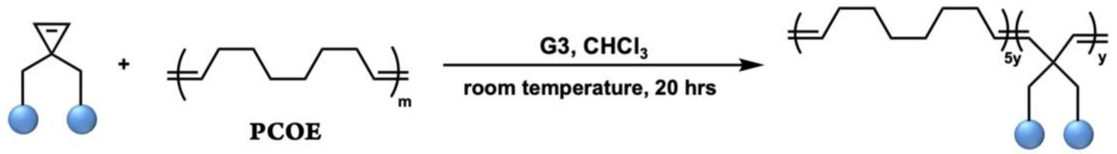


| Entry[a] | CPE | CPE/PCOE[b]/G3 | Catalyst percent (%) | [M] (mol/L) | Edited $M_n$ (kg/mol)[c] | Edited Đ[c] | Conversion (%)[d] | COE: CPE[e] |
|---|---|---|---|---|---|---|---|---|
| 1 | CPE-Bz | 40/200/0.5 | 1.2 | 0.1 | 28 | 1.57 | 34 | 14:1 |
| 2 | CPE-Bz | 40/200/0.5 | 1.2 | 0.2 | 25 | 1.99 | 49 | 9:1 |
| 3 | CPE-Bz | 40/200/0.5 | 1.2 | 0.4 | 37 | 1.82 | 62 | 8:1 |
| 4 | CPE-Bz | 40/200/0.5 | 1.2 | 0.6 | 48 | 1.80 | 60 | 7.5:1 |
| 5 | CPE-Bz | 40/200/0.5 | 1.2 | 0.8 | 41 | 1.89 | 73 | 6.4:1 |
| 6 | CPE-Bz | 40/200/0.5 | 1.2 | 1 | 66 | 1.79 | 54 | 8:1 |
| 7 | CPE-Bz | 40/200/0.5 | 1.2 | 0.8 | 48 | 1.94 | 79 | 7:1 |
| 8 | CPE-Bz | 40/200/1 | 2.4 | 0.8 | 55 | 1.74 | 93 | 5:1 |
| 9 | CPE-Bz | 40/200/2.5 | 6 | 0.8 | 32 | 1.56 | >95 | 5:1 |
| 10 | CPE-Bz | 40/200/5 | 12 | 0.8 | 19 | 1.53 | >95 | 6:1 |
| 11 | CPE-Ad | 40/200/1 | 2.4 | 0.8 | 46 | 1.75 | >95 | 6:1 |
| 12 | CPE-Py | 40/200/1 | 2.4 | 0.8 | 31 | 2.37 | 85 | 8:1 |
| 13 | CPE-PEG | 40/200/1 | 2.4 | 0.8 | 73 | 1.59 | 94 | 9:1 |

[a]Backbone editing conditions: G3 catalyst, CPE and PCOE in $CHCl_3$ at room temperature for 20 hours. [b]PCOE has a $M_n$ of 200 kg/mol and a dispersity of 1.74. [PCOE] indicates the COE units within PCOE, each COE unit has 1 olefin. [c]Number average molecular weights and dispersities after backbone editing determined by $CHCl_3$ size-exclusion chromatography (SEC) calibrated using polystyrene standards of precipitated reaction mixture. [d]Conversions were determined by $^1H$ NMR of crude reaction mixture in $CDCl_3$. [e]The ratio of COE units and CPE units are determine by $^1H$ NMR of precipitated reaction mixture.

**Table S7. Mass yield for PCOD-*co*-$CPE_{Bz}$ and PCOE-*co*-$CPE_{Bz}$.**

| Entry[a] | CPE | Polymer | CPE/Polymer/G3 | Edited $M_n$ (kg/mol)[c] | Edited $Đ$[c] | Conversion (%)[d] | monomer: CPE[e] | % mass yield |
|---|---|---|---|---|---|---|---|---|
| 1 | $CPE_{Bz}$ | PCOD | 110/550/1 | 45 | 1.65 | 94 | 6:1 | 53 |
| 2 | $CPE_{Bz}$ | PCOE | 40/200/1 | 43 | 1.79 | 92 | 5:1 | 87 |

The lower mass yield observed for PCOD-*co*-$CPE_{Bz}$ is attributed to the formation of CDT species during the backbone-editing process, which are removed during purification. In contrast, CDT formation is not observed for PCOE, resulting in a higher isolated mass yield of 87% for PCOE-*co*-$CPE_{Bz}$.

**Table S8. Thermal properties of backbone-edited polymer characterized by DSC.**

| Entry | CPE | Polymer | CPE/Polymer/G3 | [M] (mol/L) | Cyclic Monomer: CPE | $T_m$ | $T_c$ | $T_g$ |
|---|---|---|---|---|---|---|---|---|
| 1 | - | PCOD | - | - | - | 68 | 50 | - |
| 2 | CPE-Py | PCOD | 110/550/1 | 0.6 | 14:1 | 24 | -27 | - |
| 3 | CPE-PEG | PCOD | 110/550/1 | 0.6 | 6:1 | 18 | -11 | - |
| 4 | CPE-Ad | PCOD | 55/550/1 | 0.6 | 12:1 | 20 | -6 | - |
| 5 | CPE-Bz | PCOD | 55/550/1 | 0.6 | 11:1 | 21 | 3 | - |
| 6 | CPE-Py | PCOD | 27.5/550/1 | 0.6 | 25:1 | 27 | 5 | - |
| 7 | - | PCOE | - | - | - | 54 | 34 | |
| 8 | CPE-Bz | PCOE | 40/200/1 | 0.8 | 5:1 | 30 | -20 | - |
| 9 | CPE-Ad | PCOE | 40/200/1 | 0.8 | 6:1 | 30 | - | -47 |
| 10 | CPE-Py | PCOE | 40/200/1 | 0.8 | 8:1 | 40 | -22 | - |
| 11 | CPE-PEG | PCOE | 40/200/1 | 0.8 | 9:1 | 7 | 65 | -45 |

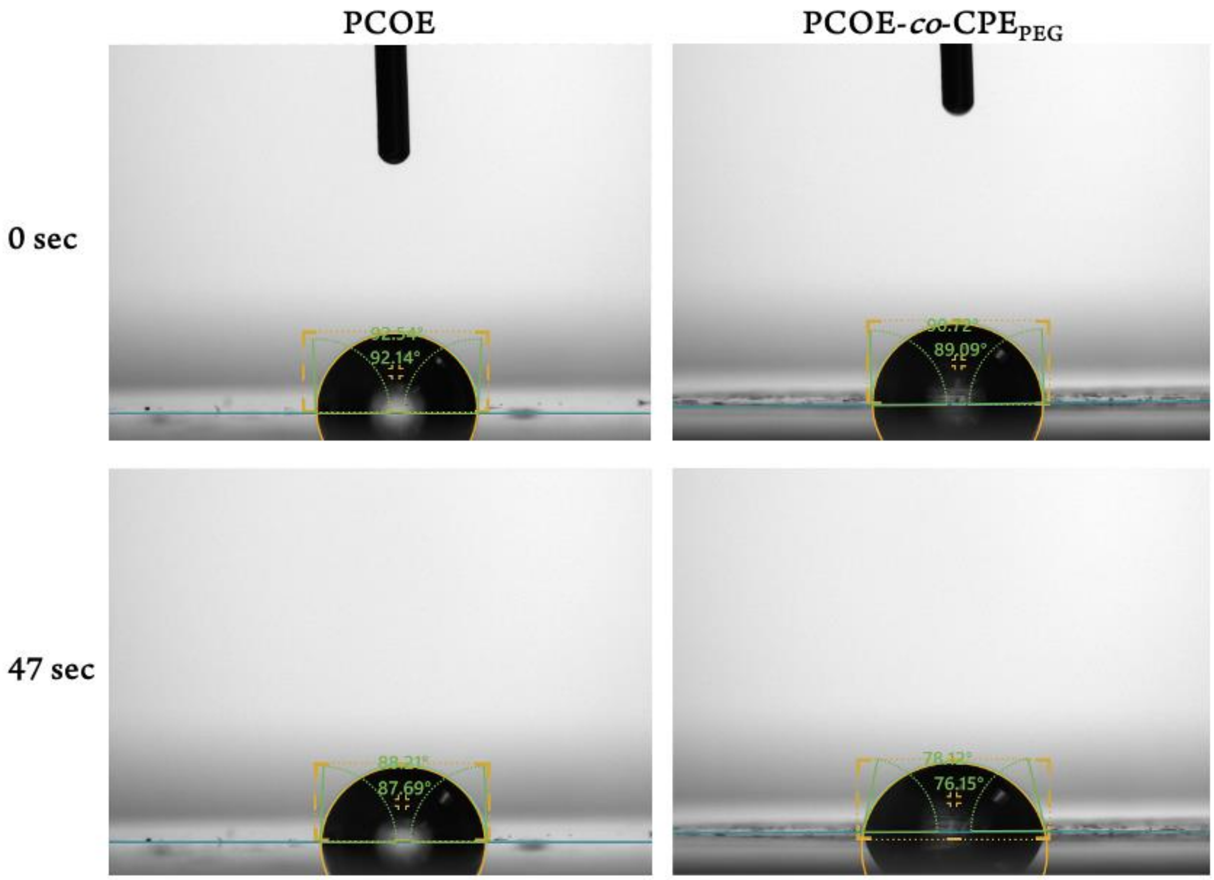


**Figure S7. Contact angle measurements on PCOE at 0 seconds (top left) and 47 seconds (bottom left) and PCOE-*co*-CPE$_{PEG}$ (PCOE to CPE$_{PEG}$ of 9:1) at 0 seconds (top right) and 47 seconds (bottom right) on polymer film made by drop casting on glass plates.**

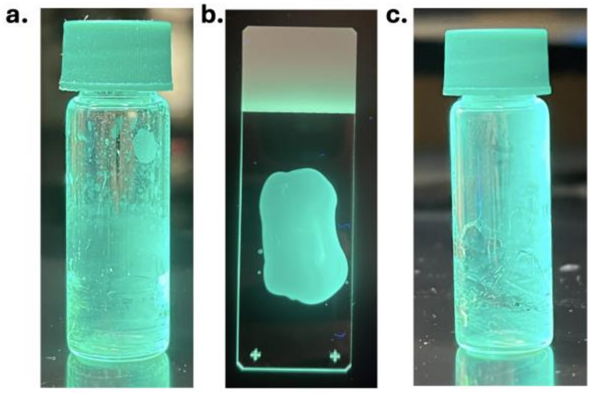


**Figure S8. a. Fluorescent properties of PCOD-*co*-CPE$_{Py}$ (Table 1, entry 8) b. PCOD-*co*-CPE$_{Py}$ fluorescent film (Table 1, entry 8), c. PCOE-*co*-CPE$_{Py}$ (Table 2, entry 3).**

## Experimental Procedures for CPE functional groups

### cycloprop-2-ene-1,1-diylbis(methylene) bis(pyrene-1-carboxylate)

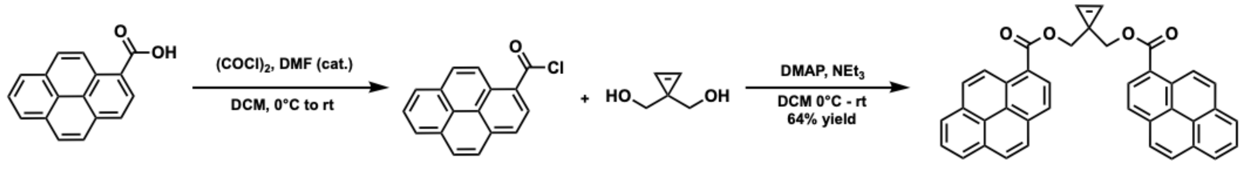


To a solution of pyrene carboxylic acid (yellow solid) (542 mg, 2.2 equiv. 2.2 mmol) in dry DCM (0.2M) at 0 °C was added oxalyl chloride (0.22 ml, 3.3 equiv. 3.3 mmol) dropwise followed by a catalytic amount of DMF (10 ul) in an oven-dried round bottom flask. Nitrogen was replaced with a deflated balloon to capture $SO_2$ and HCl. The resulting solution was stirred for 4 hours while it warmed up to room temperature and then concentrated to remove excess oxalyl chloride to give the acid chloride, which was used without further purification. The pyrene chloride dissolved in 10.5 ml dry DCM following the addition of CPE-diol (100.12 mg 1 equiv. 1 mmol) which was also dissolved in 1 ml dry DCM. Triethylamine (0.253 g, 2.5 equiv. 2 mmol) was added to an empty vial. Then, 12mg of DMAP (0.1 equiv. 0.1mmol) was dissolved in 0.5ml of DCM and added to triethylamine vial to make a solution. TEA and DMAP solution was then added to the reaction mixture dropwise at 0 °C. After the addition, ice was removed after 2 min of stirring. $N_2$ was turned to minimum to prevent DCM evaporation. The mixture was allowed to warm up to RT and stirred overnight. The mixture was concentrated in vacuo and dry load on a column. The crude mixture was purified through flash column chromatography with an eluent of hexane and ethyl acetate (7:3). The product was recrystallized in hexane for further purification, 64% yield. $^1$H NMR (400 MHz, $CDCl_3$) δ 9.17 (d, $J$ = 9.4 Hz, 2H), 8.54 (d, $J$ = 8.1 Hz, 2H), 8.10 (ddd, $J$ = 8.9, 7.5, 1.2 Hz, 4H), 8.04 (d, $J$ = 9.5 Hz, 2H), 7.99 – 7.91 (m, 4H), 7.85 (d, $J$ = 8.2 Hz, 2H), 7.78 (d, $J$ = 8.9 Hz, 2H), 7.58 (s, 2H), 4.71 (s, 4H). $^{13}$C NMR (126 MHz, $CDCl_3$) δ 168.28, 134.36, 131.26, 131.14, 130.52, 129.65, 129.51, 128.63, 127.19, 126.43, 126.40, 126.29, 125.10, 124.83, 124.25, 124.18, 123.76, 115.55, 72.11, 53.76. LCMS (m/z): calc for $C_{39}H_{24}O_4$ [M+H] 557.17 found, 557.17

### cycloprop-2-ene-1,1-diylbis(methylene) bis(adamantane-1-carboxylate)

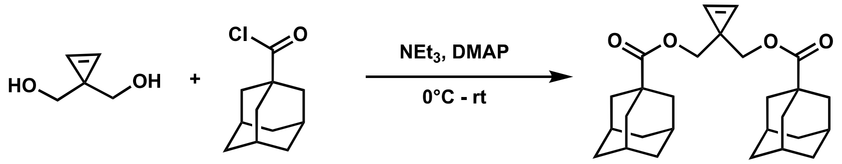


A solution of CPE-diol (0.1 g, 1 equiv. 1 mmol) in dry DCM (2.1 M) was added to DMAP (12 mg, 0.1 equiv. 0.1 mmol) under $N_2$. At 0 °C, triethylamine (0.348 ml, 2.5 equiv, 2.5 mmol) and adamantane chloride (0.437mg, 2.2 equiv, 2.2 mmol) were added sequentially. The resulting solution was warmed to rt and stirred for 14 zh. The reaction mixture was then evaporated to dryness and purified through flash column chromatography with an eluent of hexane and ethyl acetate (20:1 to 10:1). $^1$H NMR (400 MHz, $CDCl_3$) δ 7.16 (s, 2H), 4.03 (s, 4H), 2.03 (d, $J$ = 4.2 Hz, 34H), 1.99 (p, $J$ = 3.1 Hz, 7H), 1.86 (d, $J$ = 3.1 Hz, 13H), 1.75 – 1.62 (m, 14H). $^{13}$C NMR (126 MHz, $CDCl_3$) δ 177.88, 114.58, 69.20, 41.17, 39.27, 36.86, 28.31. LCMS (m/z): calc for $C_{27}H_{36}O_4$ $[M]^{\cdot+}$ 425.26 found, 425.2

## $^1$H and $^{13}$C NMR Spectra for CPE derivatives

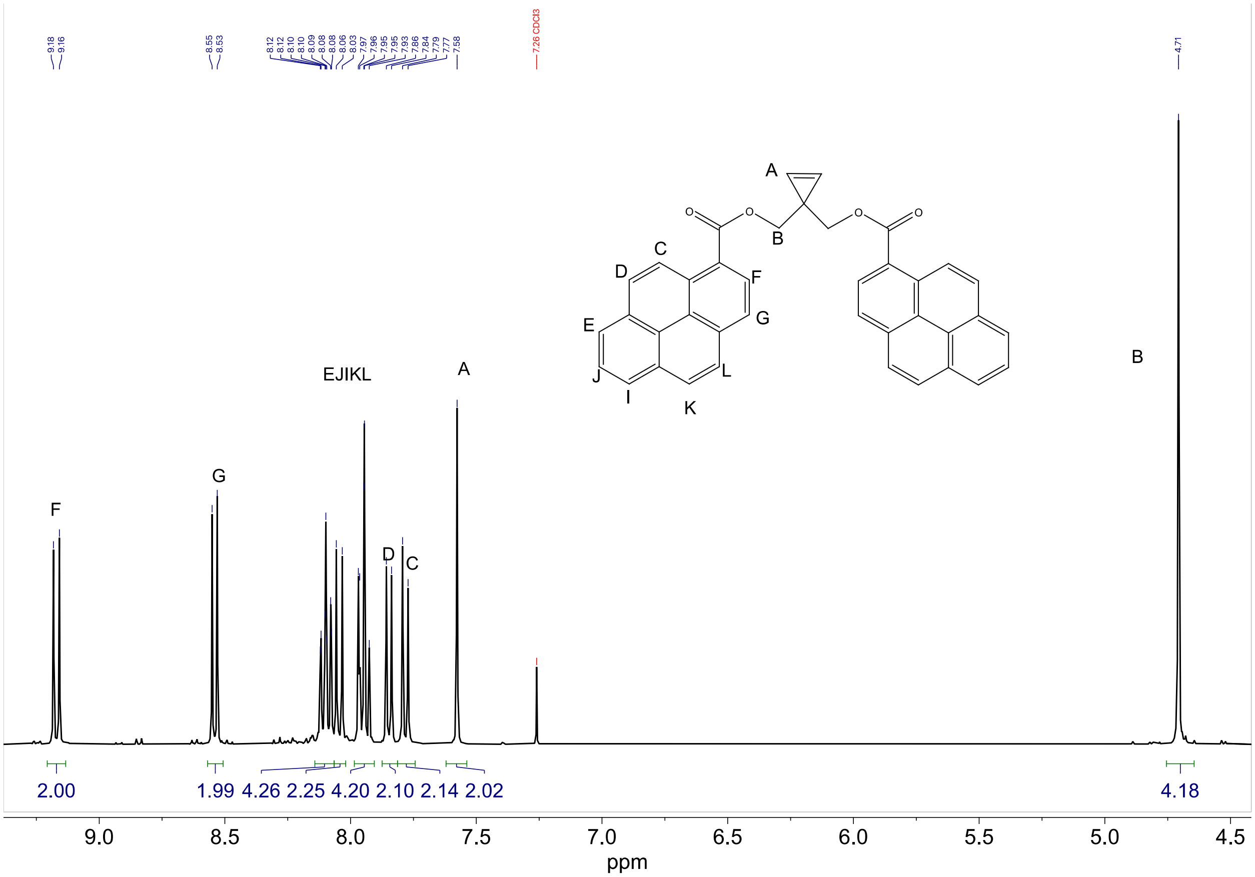


**Figure S9.** **$^1$H NMR ($CDCl_3$) of CPE-pyrene.**

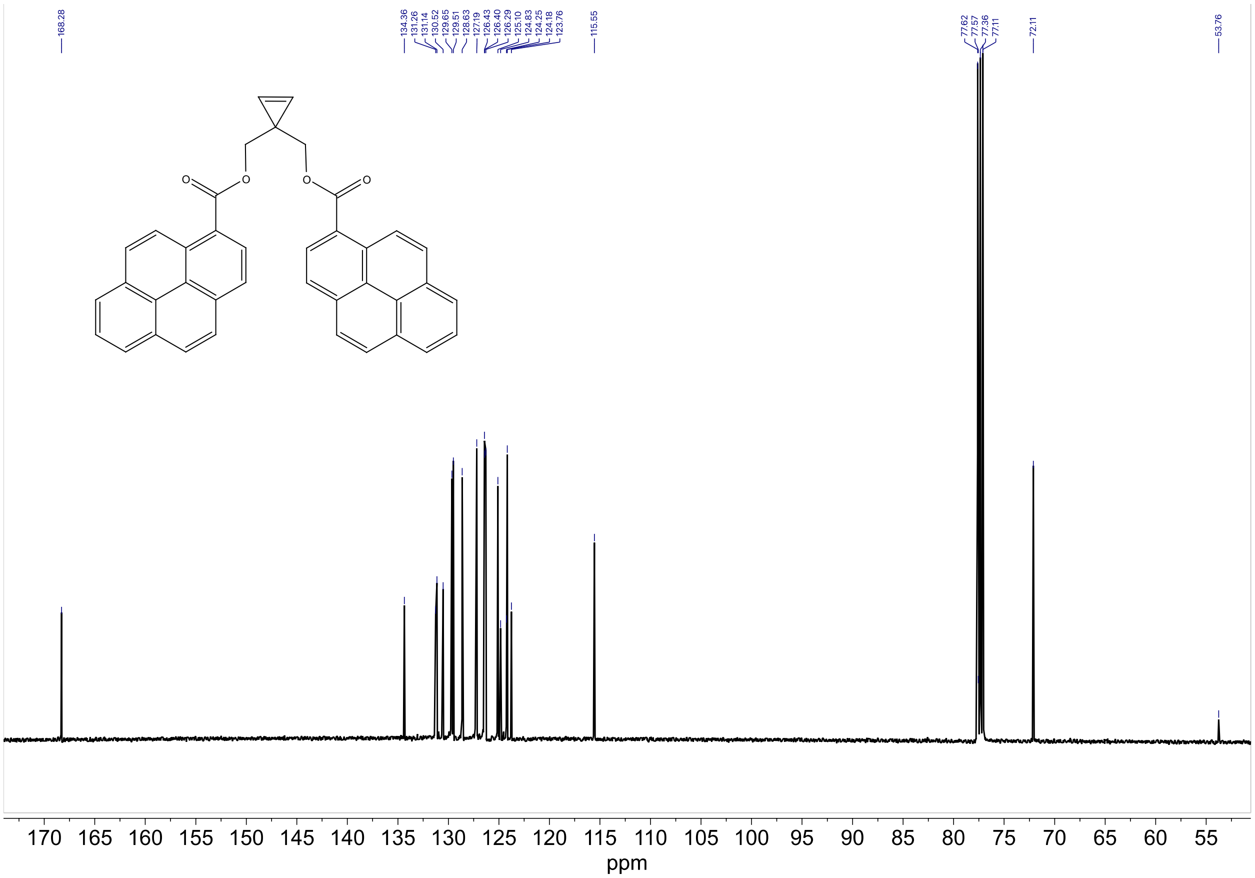

**Figure S10.** $^{13}$**C NMR (CDCl$_3$) of CPE-pyrene.**

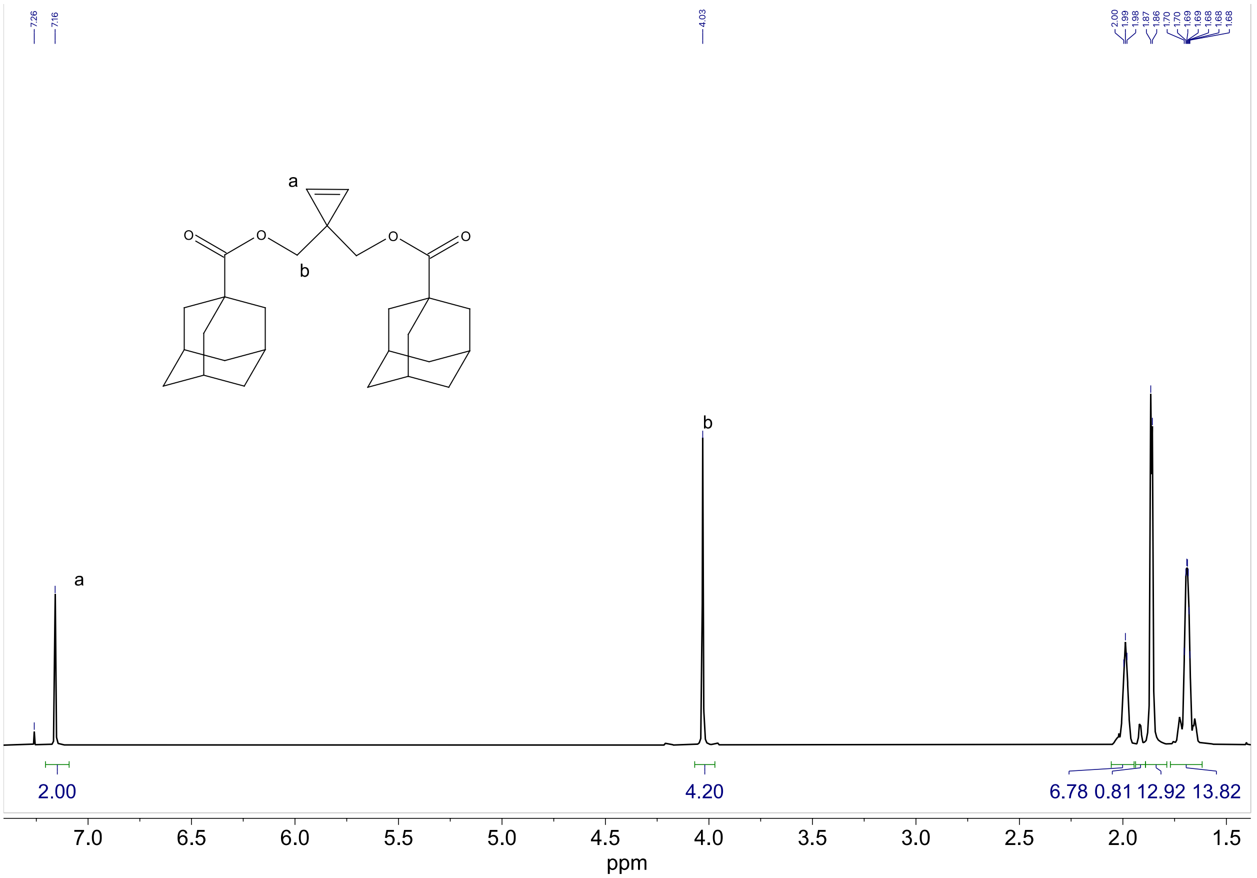


**Figure S11. $^{1}$H NMR ($CDCl_3$) of CPE-adamantane.**

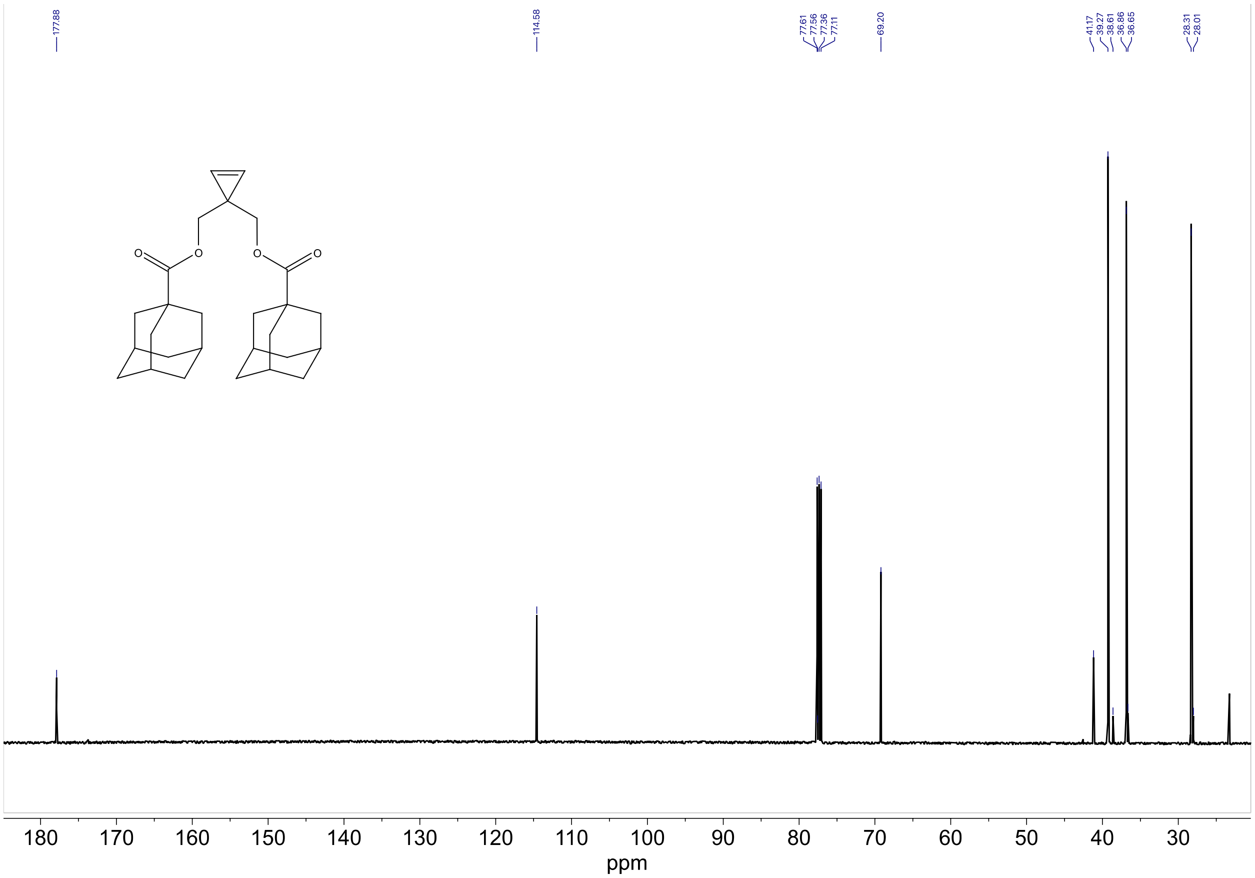


**Figure S12. $^{13}C$ NMR ($CDCl_3$) of CPE-adamantane.**

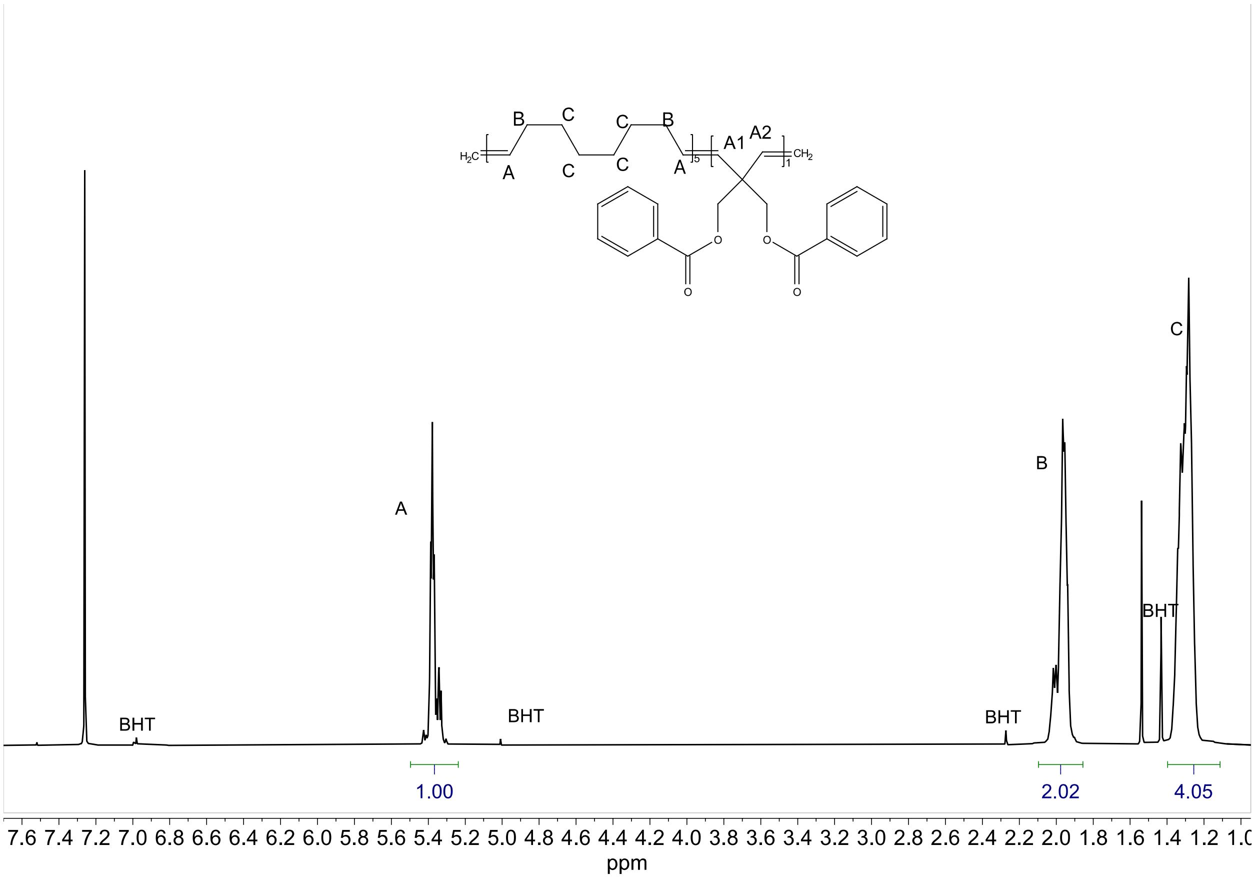


**Figure S13. $^{1}$H NMR ($CDCl_3$) of PCOE.**

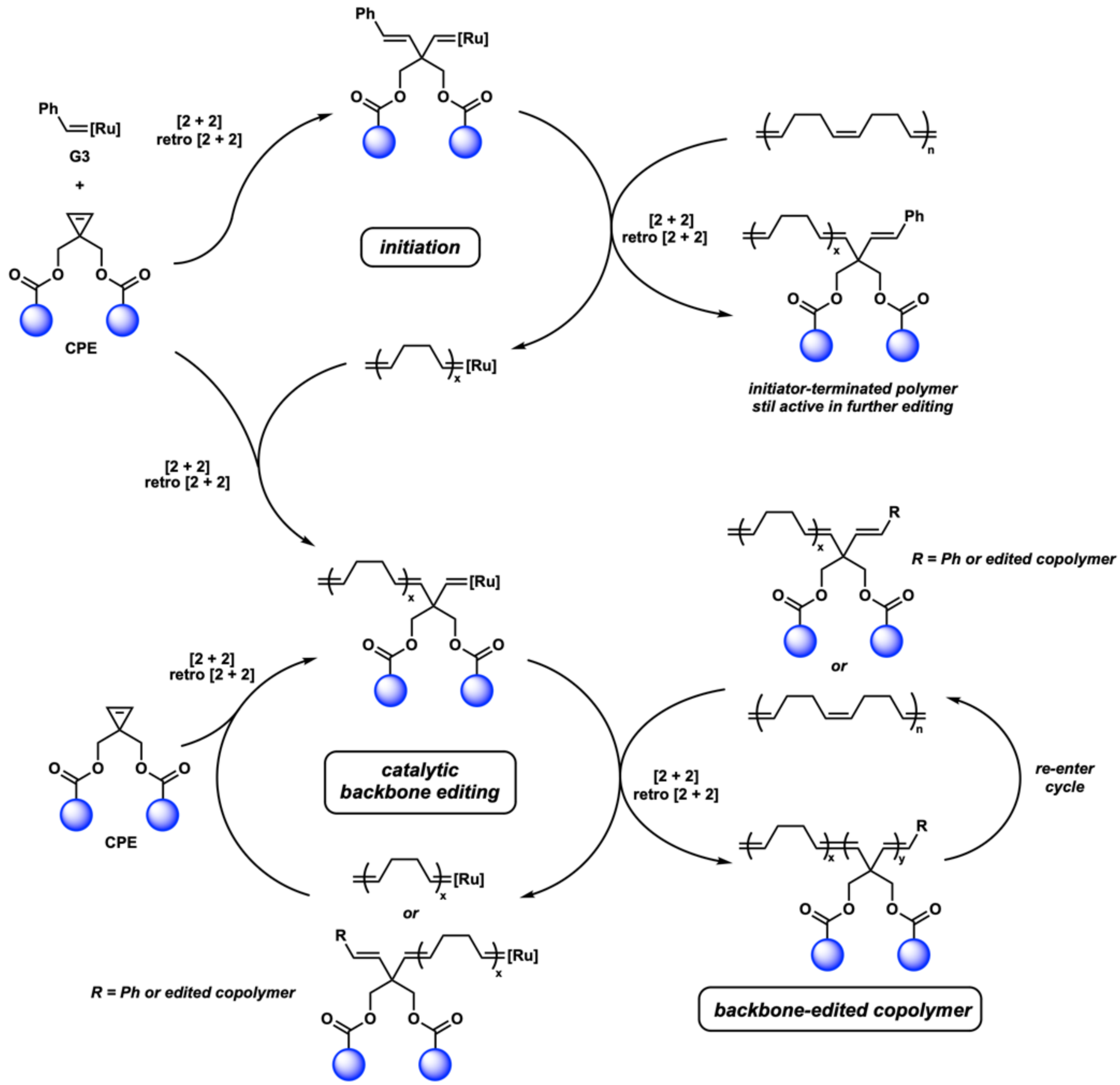


**Figure S14. Backbone-editing mechanism.**

Using Table 1, entry 1 as an example to calculate post-editing theoretical $M_n$:

$$M_n = \frac{\sum N_x M_x}{\sum N_x} = \frac{w}{N}$$

where $N_x$ is the number of moles with weight $M_x$. For ease of description, $w \equiv \sum N_x M_x$ is the total weight of all the molecules in a polymer, whereas $N \equiv \sum N_x$ is the total number of moles. If PCOD has a $M_n$ of 118,000 g/mol

$$118{,}000 \frac{\mathrm{g}}{\mathrm{mol}} = \frac{0.0250\mathrm{g}}{N} \Longrightarrow N = 2.1 \times 10^{-7} mol$$

Assuming 100% CPE added to the middle of PCOD which contributes to the increase of polymer chains

$$M_{n,\mathrm{theo}} = \frac{(\text{weight of PCOD in the reaction}) + (\text{weight of CPE in the reaction})}{(\text{mmol of PCOD} + \text{percentage of G3 according to CPE} \times \text{mmol of CPE}}$$
$$= \frac{(0.025\mathrm{g} + 0.014\mathrm{g})}{(0.00021\mathrm{mmol} + 0.91\% \times 0.046\mathrm{mmol})} = 62.0\mathrm{kg/mol}$$

Experimentally, the precipitated backbone-edited polymer has 6:1 PCOD units:CPE-OBz. With the secondary metathesis leading to the formation of CDT as a side product that contributes to the loss of PCOD in the backbone editing process, the experimental $M_n$ of the edited polymer is 39 kg/mol. From the crude backbone editing mixture, CDT:CPE ratio is 0.36:1

$$Weight\ of\ CDT = 0.046mmol \times 0.36 \times 162.28 \frac{mg}{mmol} = 2.69\ mg$$

$$CPE\ weight\ on\ the\ edited\ PCOD = \frac{(25\ mg - 2.69\ mg)}{108.18 \frac{mg}{mmol}} \times \frac{1}{6} \times \frac{308.33\ mmol}{mg} = 10.60\ mg$$

$$PCOD\ weight\ on\ the\ edited\ PCOD = 25\ mg - 2.69\ mg = 22.31\ mg$$

$$Total\ weight\ of\ the\ CPEBz\ edited\ PCOD = 22.31 + 10.6 = 32.91\ mg$$

$$M_{n,\mathrm{experimental}} = \frac{(32.91\ mg)}{(0.00021\mathrm{mmol} + 0.91\% \times 0.046\mathrm{mmol})} = 52.4\ \mathrm{kg/mol}$$

**Figure S15. Backbone-editing mechanism.**

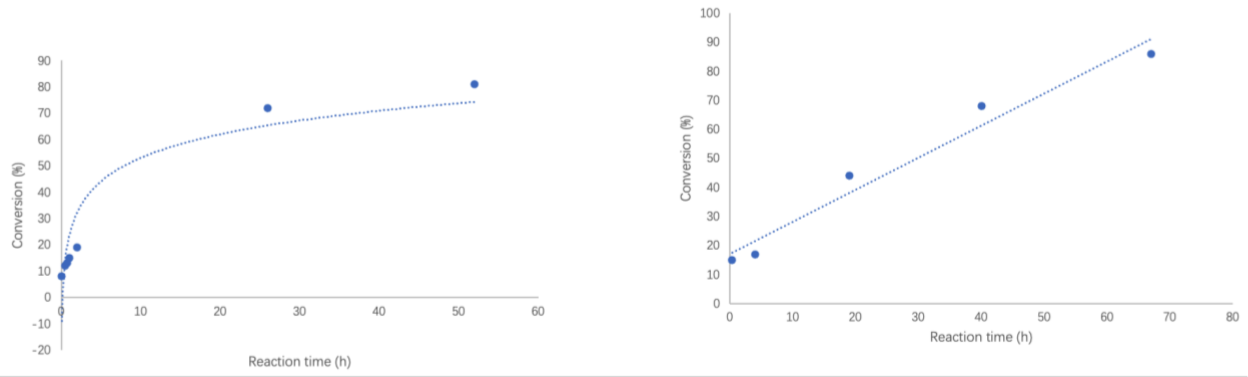


**Figure S16. Kinetics of CPE-Bz PCOD backbone editing (left) and CPE-Bz PCOE backbone editing (right) in $CDCl_3$.**

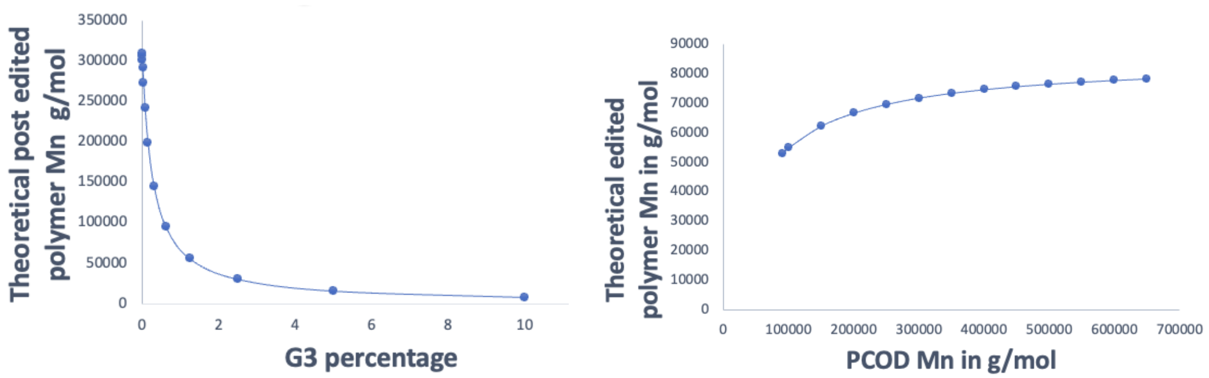


**Figure S17. Impact on 200 kDa PCOD using different quantities of catalyst (Left). Impact on varying pre-edited Mn of PCOD with 1% G3 (Right).**

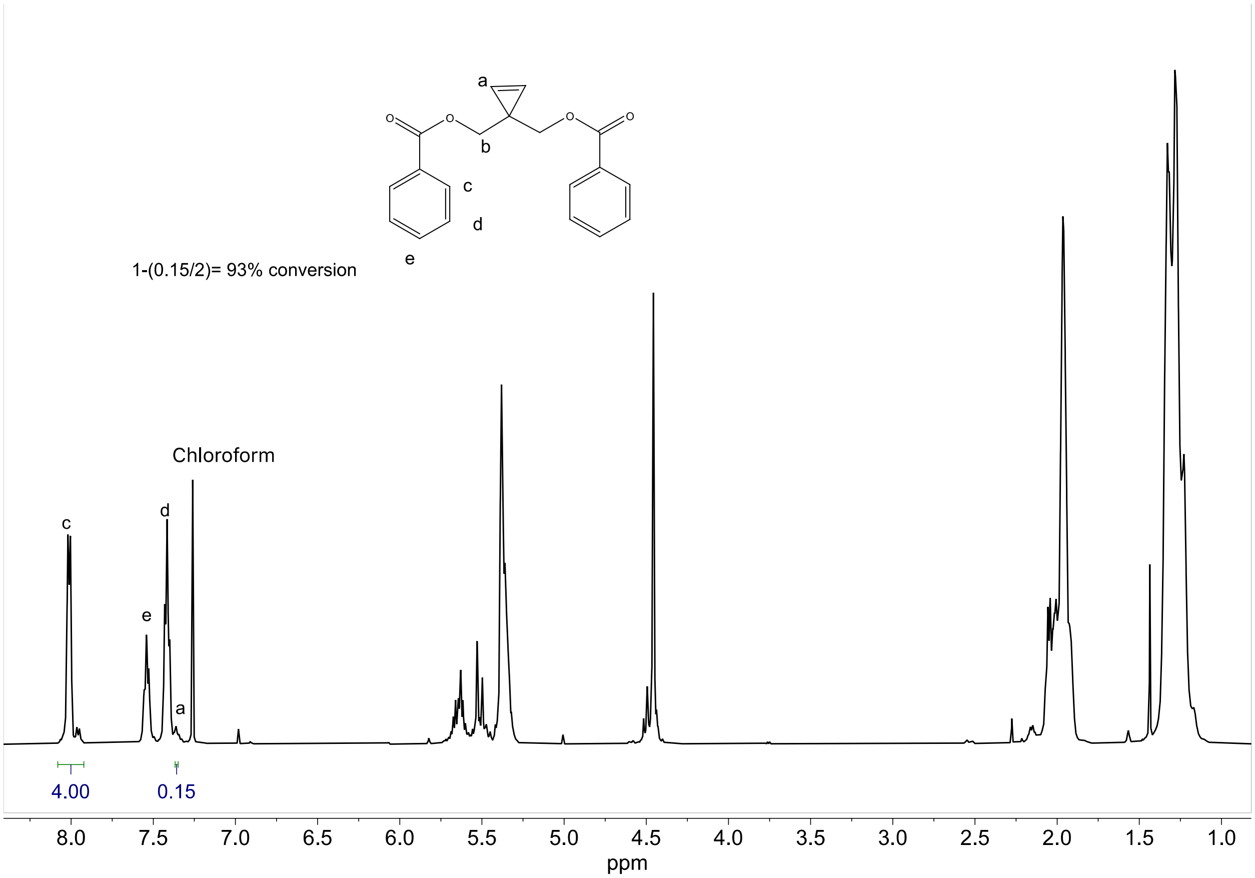


**Figure S18. Crude $^{1}$H NMR spectra for CPE-Bz edited PCOE conversion calculations.**

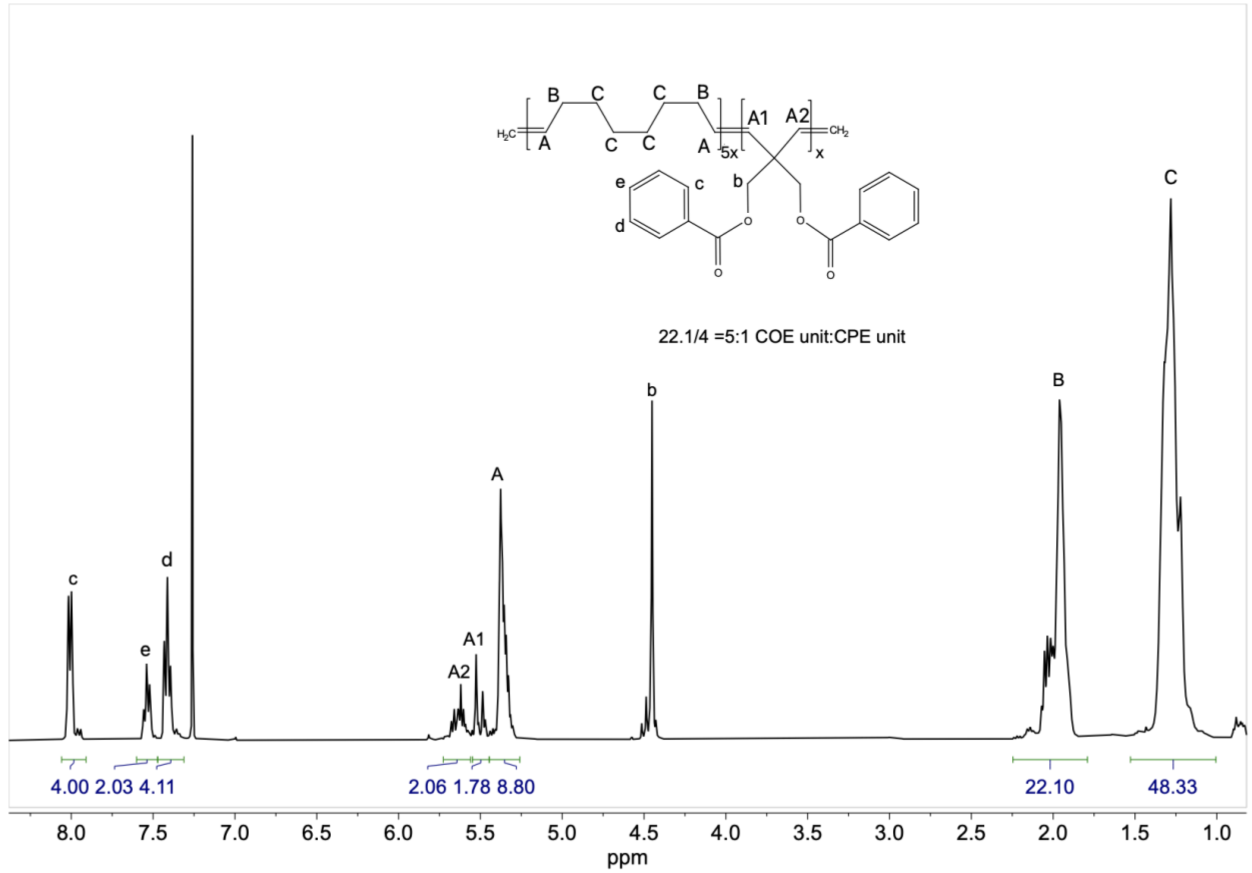


**Figure S19. Pure $^{1}$H NMR spectra for CPE-Bz edited PCOE conversion and COE:CPE-Bz calculations.**

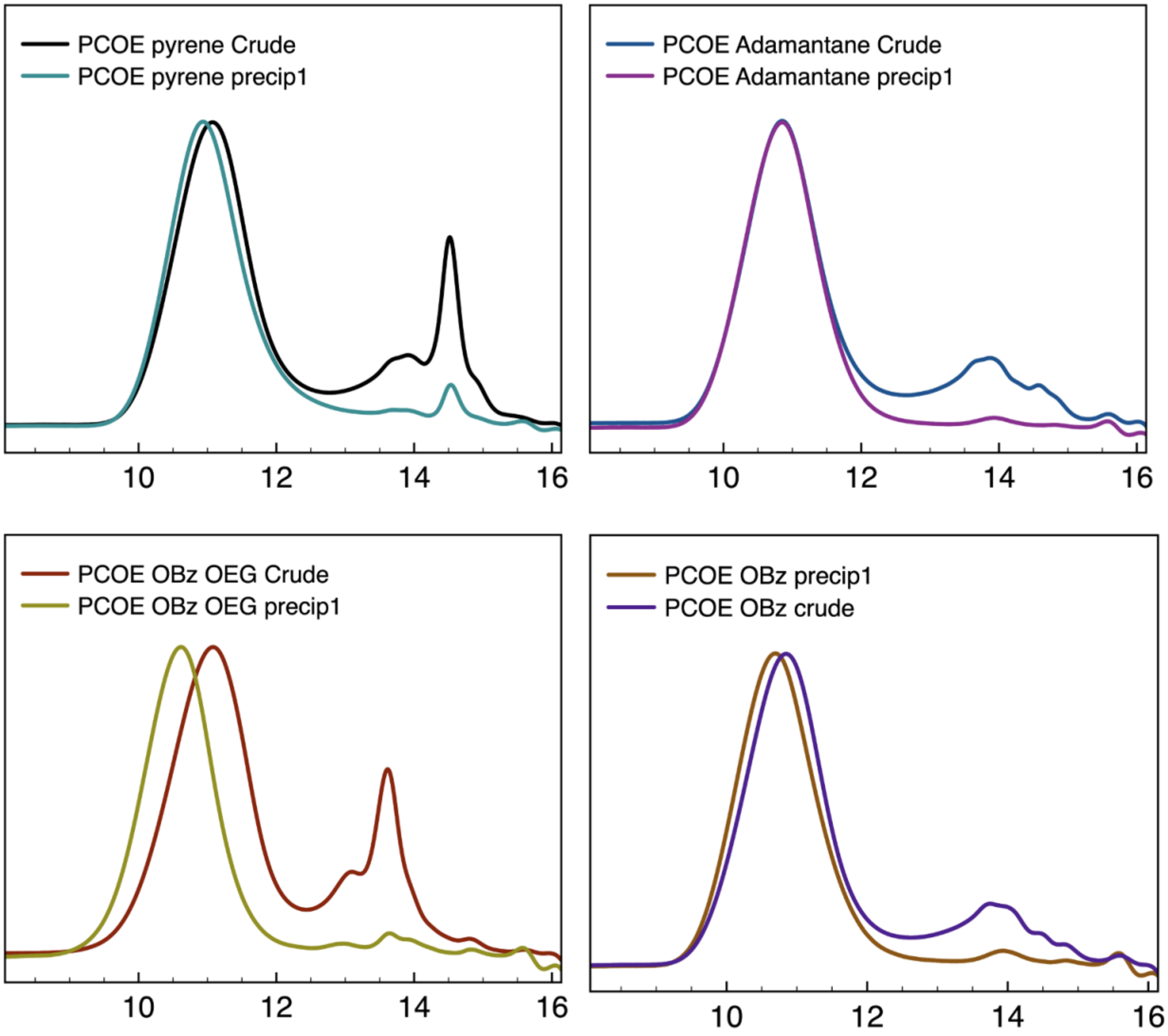


**Figure S20. GPC of PCOE backbone editing crude and precipitated polymer comparison (SEC $CHCl_3$).**

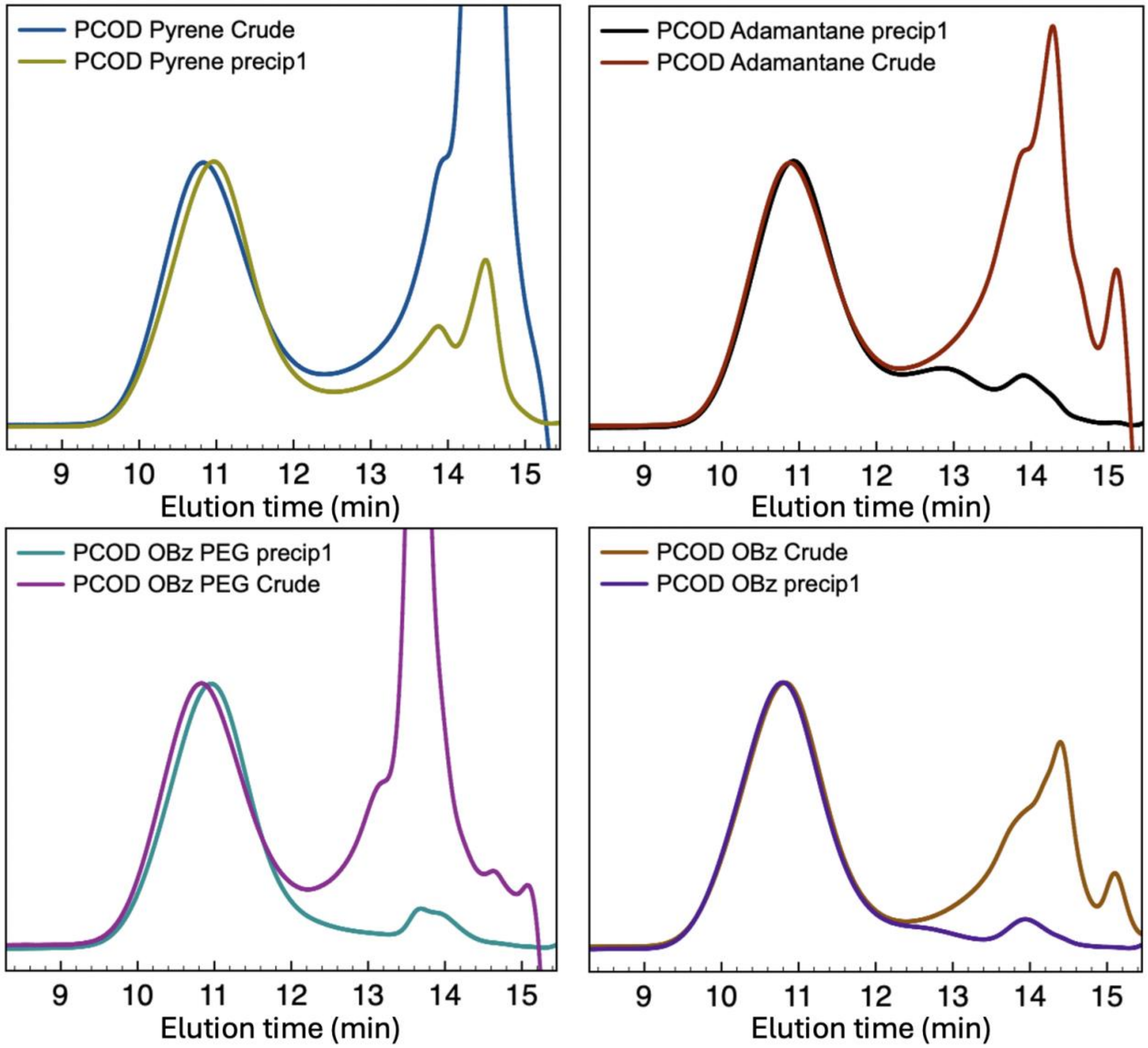


**Figure S21. GPC of PCOD backbone editing crude and precipitated polymer comparison.**
